# Aberrant Molecular Myelin Architecture in Charcot–Marie–Tooth Disease Type 1A and Hereditary Neuropathy With Liability to Pressure Palsies

**DOI:** 10.1002/glia.70124

**Published:** 2025-12-16

**Authors:** Kathryn R. Moss, Marvis A. Arowolo, Dave R. Gutierrez, Ahmet Höke

**Affiliations:** ^1^ Department of Physical Medicine and Rehabilitation University of Missouri School of Medicine Columbia Missouri USA; ^2^ NextGen Precision Health University of Missouri Columbia Missouri USA; ^3^ Department of Neurology, Neuromuscular Division Johns Hopkins University School of Medicine Baltimore Maryland USA; ^4^ Department of Neuroscience Johns Hopkins University School of Medicine Baltimore Maryland USA

**Keywords:** Charcot–Marie–Tooth disease (CMT), Charcot–Marie–Tooth disease type 1A (CMT1A), hereditary neuropathy with liability to pressure palsies (HNPP), microscopy, myelin, neuromuscular disease, node of Ranvier, peripheral myelin protein 22 (PMP22), Schmidt‐Lanterman incisure (SLI), Schwann cell

## Abstract

Charcot–Marie–Tooth Disease Type 1A (CMT1A) and Hereditary Neuropathy with Liability to Pressure Palsies (HNPP) are the most common inherited peripheral neuropathies and arise from copy number variation of the Peripheral Myelin Protein 22 (*PMP22*) gene. While secondary axon degeneration has been proposed as the primary driver of disability, our prior work demonstrated pronounced neuromuscular impairment in CMT1A model mice in the absence of overt axonal loss, prompting investigation into primary myelin dysfunction. Here, we reveal that altered *PMP22* dosage profoundly disrupts molecular architecture at critical myelin domains, Schmidt‐Lanterman incisures (SLIs) and Nodes of Ranvier. Using high‐resolution confocal imaging of teased peripheral nerve fibers from CMT1A and HNPP model mice, we identified widespread disorganization of adherens junctions, mislocalization of Connexin29 and aberrant distribution of nodal ion channels, with several defects more severe in CMT1A, consistent with disease burden. Notably, nodal widening and abnormal spreading of Kv1.2 and Caspr along internodes indicate compromised axo‐glial compartmentalization essential for saltatory conduction. Together, these findings support a model in which *PMP22* functions as a structural organizer of myelin, coordinating adherens junction patterning and nodal subdomain integrity. Dysregulation of this function is predicted to compromise Schwann‐cell architecture, metabolic support and axonal excitability. Our findings support a paradigm shift in which molecular destabilization of myelin, rather than secondary axonal degeneration alone, contributes to disease progression in CMT1A and HNPP. This work also identifies junctional complexes as potential actionable molecular targets and establishes a mechanistic framework applicable to a broad spectrum of inherited dysmyelinating and acquired demyelinating neuropathies.

## Graphical abstract

- *PMP22* copy number variation disrupts myelin architecture at SLIs and Nodes of Ranvier.
- Adherens junction and axoglial domain defects are often more severe in CMT1A than HNPP.
- Findings support PMP22 functioning as a structural organizer of myelin.

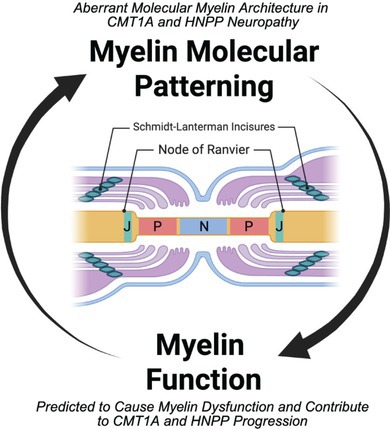

**Abbreviations**
CADM4: Cell Adhesion Molecule 4
CMT: Charcot–Marie–Tooth Disease
CMT1A: CMT Type 1A
CMT1E: CMT Type 1E
CMT1X: CMT Type 1X
Cx29: Connexin29
Cx32: Connexin32
ECL1: extracellular loop 1
HNPP: Hereditary Neuropathy with Liability to Pressure Palsies
MAG: Myelin‐Associated Glycoprotein
P: postnatal day
PMP22: Peripheral Myelin Protein 22
SLIs: Schmidt‐Lanterman Incisures
WT: wildtype

## 1 Introduction

Charcot–Marie–Tooth Disease Type 1A (CMT1A) and Hereditary Neuropathy with Liability to Pressure Palsies (HNPP) are the most common inherited peripheral neuropathies, together accounting for approximately 65% of genetically defined CMT cases (Li et al. 2013). The overall prevalence of CMT is estimated at 1 in 2500 individuals, making it one of the most prevalent inherited neurological conditions (Martyn and Hughes 1997). Remarkably, CMT1A and HNPP result from opposing copy number variations of the same gene: *Peripheral Myelin Protein 22* (*PMP22*), which encodes PMP22 protein. Duplication of *PMP22* causes CMT1A, a progressive dysmyelinating neuropathy that typically presents in childhood and leads to steadily worsening motor and sensory deficits, often resulting in lifelong disability (Watila and Balarabe 2015). In contrast, deletion of *PMP22* causes HNPP, a milder disorder characterized by transient, focal neuropathies triggered by mechanical compression, with onset typically in adolescence or adulthood (Attarian et al. 2020). Although recovery is common in HNPP, repeated episodes can cause permanent nerve damage (Attarian et al. 2020). Despite differences in severity and progression, both conditions are associated with significant morbidity, and no disease‐modifying therapies currently exist (Park et al. 2020; Vaeth et al. 2017).

PMP22 is a Schwann cell‐enriched membrane protein whose precise physiological function remains incompletely defined (Vallat et al. 1996). While CMT1A and HNPP are classified as primary myelin disorders, the prevailing pathogenic model emphasizes secondary axonal degeneration as the driver of clinical deficits (Krajewski et al. 2000; Manganelli et al. 2016). However, despite severe motor impairments, reduced compound muscle action potentials and significant muscle atrophy observed in CMT1A mouse models (Moss et al. 2022; Verhamme et al. 2011), we and others have reported no clear evidence of denervation or axonal loss (Moss et al. 2022; Robertson et al. 2002). These findings implicate primary myelin dysfunction as a central contributor to disease pathogenesis and highlight the need to better understand the structural and molecular consequences of PMP22 dysregulation in myelin.

PMP22 is structurally related to Claudins, a family of tight junction proteins (pfam00822) that mediate intercellular adhesion and membrane compaction (Simske 2013; Van Itallie and Anderson 2006). Despite limited sequence identity (~25%), AlphaFold‐predicted structures reveal strong topological similarity between PMP22 and Claudin‐1, particularly across the first extracellular loop (ECL1), suggesting a potentially conserved role in adhesion. Supporting this idea, PMP22 has been demonstrated to localize to cell borders with adhesion junctions in epithelial and endothelial cells, where its overexpression increases transepithelial electrical resistance (Notterpek et al. 2001; Roux et al. 2004, 2005). In peripheral nerve, PMP22 localizes to compact myelin and has been implicated in the organization of both tight junctions and adherens junctions. Prior studies have shown that key junctional components, including Claudin‐19, ZO‐1, ZO‐2, JAM‐C, and E‐Cadherin, are disrupted in PMP22‐deficient mice (Guo et al. 2014; Hu et al. 2016; Neuberg et al. 1999). In PMP22 null mice (*PMP22* ^−/−^), E‐Cadherin becomes abnormally dispersed along internodes rather than enriched at Schmidt‐Lanterman incisures (SLIs) (Neuberg et al. 1999) and approximately 15% of SLIs in 3‐month‐old HNPP model mice (*PMP22* ^+/−^) were qualitatively reported to exhibit abnormal E‐Cadherin immunostaining (Hu et al. 2016). In the same mouse model, Claudin‐19 exhibited reduced and asymmetric accumulation near Nodes of Ranvier (Guo et al. 2014), and about 5% of SLIs were reported to be abnormal for MAG at 3 months (Hu et al. 2016). While these qualitative observations implicated PMP22 in junctional integrity, the magnitude and direction of these alterations, particularly across CMT1A and HNPP, have not been quantitatively defined.

To more precisely determine whether PMP22 regulates adhesion and how gene dosage impacts myelin integrity in CMT1A and HNPP models, we systematically analyzed teased peripheral nerve fibers using high‐resolution confocal imaging. Unlike prior qualitative assessments, which described junctional abnormalities in categorical terms, our study provides the first quantitative characterization of these changes using two independent metrics, distribution (signal volume normalized to fiber diameter) and mean fluorescence intensity, enabling direct comparison between CMT1A, HNPP, and wildtype (WT) myelin. Our data reveal that altered *PMP22* dosage leads to marked disruptions in adherens junction components, including E‐Cadherin, β‐Catenin, and F‐Actin, which are prominently localized to SLIs. These disruptions were more pronounced in CMT1A, where SLIs appeared abnormally compacted relative to WT nerves. In contrast, nerves from HNPP model mice exhibited more dispersed staining at SLIs, particularly evident with the distribution of F‐Actin, while E‐Cadherin and β‐Catenin showed more modest changes. These findings suggest that *PMP22* dosage inversely affects adherens junction organization and likely SLI morphology. Importantly, not all SLI‐resident proteins were affected in the same manner. We also examined additional adhesion‐related proteins, including Cell Adhesion Molecule 4 (CADM4), Connexin29 (Cx29), Myelin‐Associated Glycoprotein (MAG), and Connexin32 (Cx32). While CADM4 distribution remained unchanged in both CMT1A and HNPP, Cx29 and MAG displayed distinct alterations compared to adherens junction components. Specifically, both proteins exhibited increased distributions and increased mean signal intensity at SLIs in CMT1A and HNPP nerves, with changes generally more pronounced in CMT1A. In contrast, Cx32 demonstrated reduced mean signal intensity at SLIs in both CMT1A and HNPP. These findings highlight selective and protein‐specific molecular changes at SLIs that likely influence the structure and function of this specialized compartment.

Given the anatomical and functional link between SLIs and the Node of Ranvier via the inner mesaxon (Scherer and Arroyo 2002) and prior studies showing that Cx29 associates with Kv1 channels at juxtaparanodes (Rash et al. 2016), we next examined the molecular organization of Nodes of Ranvier. All three nodal subdomains were affected, with changes typically correlating with disease severity: CMT1A nerves showing more pronounced alterations than HNPP. At the node, voltage‐gated sodium channels (Nav) exhibited evidence of nodal widening. At juxtaparanodes and paranodes, Kv1.2 and Caspr, respectively, demonstrated abnormal distributions including spreading along the internode. Therefore, our findings support a model in which PMP22 functions as a structural organizer of myelin architecture, coordinating adherens junction organization and nodal subdomain patterning. Disruption of this function is predicted to destabilize Schwann‐cell architecture prior to demyelination, impair metabolic coupling and compromise axonal excitability. This work challenges the long‐standing axon‐centric paradigm driving functional decline in CMT1A and HNPP and identifies junctional complexes as potential actionable molecular targets for therapeutic intervention.

## 2 Materials and Methods

### 2.1 Animal Husbandry

All experiments were conducted with approval from the Johns Hopkins University and University of Missouri Animal Care and Use Committees. C3‐PMP mice (B6.Cg‐Tg(PMP22) C3Fbas/J, referred to as CMT1A model mice) were obtained from the Jackson Laboratory (Stock #: 030052), maintained/expanded as heterozygotes by breeding with C57BL/6J WT mice (the Jackson Laboratory, Stock #: 000664) and genotyped according to the suggested protocol. LacZ PMP22 Deficient mice (referred to as HNPP model mice) were obtained from Regeneron via Dr. Lucia Notterpek, maintained/expanded as heterozygotes by breeding with 129S1/SvImJ WT mice (the Jackson Laboratory, Stock #: 002448) and genotyped according to the suggested protocol (Adlkofer et al. 1995). A balanced representation of male and female mice aged 3 months or postnatal day 15 (P15) were perfused and sciatic nerves were harvested to prepare teased nerve fibers for immunostaining.

### 2.2 Perfusion and Teased Nerve Fibers

Mice were anesthetized with Isoflurane and transcardiac perfusion was performed under continuous anesthesia. Blood was cleared by perfusion with chilled 1× PBS followed by perfusion with chilled 4% paraformaldehyde prepared in 1× PBS. After perfusion, both sciatic nerves were dissected and post‐fixed in 4% paraformaldehyde for 3 h at 4°C. The nerves were then washed five times quickly with cold 1× PBS and stored at 4°C overnight. The next day, the tibial branch was isolated in a dish of cold 1× PBS and cut into four segments. Each segment was transferred to a drop of 1× PBS on a Superfrost Plus microscope slide (Fisher Scientific) and gently teased with fine needles to separate the nerve fibers. After teasing, excess PBS was removed and the teased nerve fiber slides were dried at room temperature overnight. The slides were stored at −80°C the next day until ready for immunofluorescence staining.

### 2.3 Immunofluorescence Staining of Teased Nerve Fibers

Teased nerve fibers for immunofluorescence were permeabilized by submerging the slides in −20°C chilled acetone in Coplin jars for 10 min while rocking at room temperature. After removing the acetone, fibers were washed with 1× PBS three times for 5 min each while rocking at room temperature. The edges of each slide were quickly dried and a boundary around the fibers was drawn using a PAP pen. This was performed one slide at a time to prevent drying of the nerve fibers. Slides were resubmerged in the last 1× PBS wash after the PAP boundary was dry. Excess 1× PBS was removed from each slide before placing the slides into an immunostaining tray and adding 0.5 mL of antibody buffer per slide (190 mL—1× PBS, 10 mL—40%–50% fish gelatin [Sigma Aldrich, G7765] and 200 μL Triton X‐100, aliquots stored at −20°C). This was performed in batches of three slides to prevent drying of the nerve fibers. Fibers were blocked with antibody buffer at room temperature for 1 h. Primary antibody solutions (see Table 1 for concentrations) were prepared in antibody buffer on ice. After removing the antibody buffer used for blocking, 0.5 mL of primary antibody solution was added to each slide and incubated at 4°C overnight.

**TABLE 1:** Primary antibodies used in this study.

Primary antibody	Company, Cat. number	Species	Concentration
Anti‐E‐cadherin	Thermo Fisher, 14‐3249‐82	Rat	1:100
Anti‐neurofascin155 (Pan)	R&D Systems, AF3235	Chicken	1:500
Anti‐ZO‐1	DSHB, R26.4C	Rat	1:50
Anti‐β‐catenin	Millipore Sigma, C2206	Rabbit	1:500
Anti‐p120‐catenin	Thermo Fisher, PA5‐82545	Rabbit	1:500
Anti‐CADM4	Thermo Fisher, MA5‐24141	Rat	1:100
Anti‐connexin29	Thermo Fisher, 34‐4200	Rabbit	1:50
Anti‐connexin32	Thermo Fisher, 34‐5700	Rabbit	1:50
Anti‐Nav (pan)	Absolute antibody, Ab02113‐8.1	Rat	1:500
Anti‐Kv1.2	Absolute antibody, Ab02104‐23.0	Rabbit	1:500
Anti‐Caspr	Abcam, ab34151	Rabbit	1:1000
Anti‐βIII‐tubulin	Millipore Sigma, T2200	Rabbit	1:500

The next day, nerve fibers were washed with 1× PBS three times for 10 min each while rocking at room temperature. Secondary antibody solutions (see Table 2 for concentrations) were prepared in antibody buffer on ice. Excess 1× PBS was removed from each slide before placing the slides into an immunostaining tray and adding 0.5 mL of secondary antibody solution to each. This was performed in batches of three slides to prevent drying of the nerve fibers. The nerve fibers were incubated with secondary antibody at room temperature for one and a half hours.

**TABLE 2:** Secondary and direct conjugated antibodies used in this study.

Secondary or direct conjugated antibody	Company	Concentration
405 Phalloidin	Thermo Fisher, A30104	1:400
MAG Alexa Fluor 488	Sigma‐Aldrich, MAB1567A4	1:1000
Goat anti‐Rabbit Alexa Flour 488	Thermo Fisher, A11034	1:1000
Goat anti‐Rabbit Alexa Flour 647	Thermo Fisher, A21245	1:1000
Goat anti‐Rat Alexa Fluor 568	Thermo Fisher, A11077	1:1000
Goat anti‐Chicken Alexa Fluor 647	Thermo Fisher, A11041	1:1000

The nerve fibers were washed with 1× PBS three times for 10 min each while rocking at room temperature.

Excess 1× PBS was removed, 60 μL Prolong Gold antifade reagent (Thermo Fisher, P36930) was added to each and slides were topped with No. 1.5H Precision cover glass. Slides were dried at room temperature overnight in the dark and stored at −20°C the next day until ready for imaging.

### 2.4 Confocal Immunofluorescence Imaging, Data Analysis, and Statistics

Confocal imaging was performed using a Zeiss LSM800, Zeiss LSM880, or Olympus FV4000 with a 60× oil objective. Z‐stacks were acquired from at least six different regions per mouse per condition and analyzed using Imaris ×64 version 9.2.1 (Oxford Instruments, Bitplane). Five SLIs from at least three different nerve fibers were manually outlined in each image to create isosurfaces. Mean signal intensity was measured within each SLI isosurface. The signal for a channel of interest was then duplicated only within each SLI isosurface (masked channel) and a threshold was established as real signal for each antibody and kept constant throughout. A second isosurface was created using the masked channel by setting the established signal threshold. The volume of the second isosurface and the diameter of the nerve fiber near each analyzed SLI (axon + myelin sheath) were recorded. The distribution at SLIs was calculated by normalizing each thresholded signal volume to the corresponding fiber diameter.

Nodes of Ranvier, generally from two to three different nerve fibers, were analyzed similarly by manually tracing a node and the adjacent internodal myelin sheaths on both sides of the node up until the first SLI or a cross point with another nearby myelinated fiber. Mean signal intensity was measured within each node + proximal internode isosurface. The signal for a channel of interest was then duplicated only within each node + proximal internode isosurface (masked channel) and a threshold was established as real signal for each antibody and kept constant throughout. A second isosurface was created using the masked channel by setting the established signal threshold. The volume of the second isosurface and the diameter of the nerve fiber (axon + myelin sheath) were recorded. The distribution at Nodes of Ranvier was calculated by normalizing each thresholded signal volume to the fiber diameter. Internodal spread for Kv1.2 and Caspr was measured by selecting only the second isosurfaces near the node using the circle selection tool in Imaris and subtracting the volume at the node from the total volume and normalizing this to the total node + proximal internode isosurface volume.

For qualitative segmental demyelination analysis, tiled widefield imaging was performed using an Olympus IX83 with a 60× oil objective. G‐ratios were calculated from confocal images by measuring axon diameters in the Phalloidin/F‐Actin channel after adjusting the lookup table to visualize axonal boundaries. These values, together with fiber diameters (axon + myelin) previously measured for distribution analyses, were used to calculate G‐ratio (axon diameter/fiber diameter). Images from the same datasets used for SLI and nodal distribution analyses were used for these measurements.

All data were evaluated for significance using Prism 9 (GraphPad). For each experiment, the data were analyzed by three statistical tests: (1) unpaired *t*‐test with all individual data points, (2) unpaired *t*‐test with experimental means (*n* = 5) and (3) nested *t*‐test comparing individual data points grouped by experiment. Significance detected (*p* < 0.05) with all three *t*‐test statistics is denoted as ***, with only two of these *t*‐test statistics (tests #1 and #2) denoted as ** and with only one (test #1) denoted as *. Fold changes, *p*‐values for the most stringent statistical test demonstrating significance, exact n, definition of what n represents, and graphical display details are located in the results and figure legends. Randomization was performed for all experiments by randomly selecting mice from multiple litters and representing both sexes in each cohort. No sex differences were detected and no data points from any experiment were excluded from this study.

## 3 Results

### 3.1 Structural Homology to Claudins Points to a Role for PMP22 in Adhesion

**FIGURE 1:**
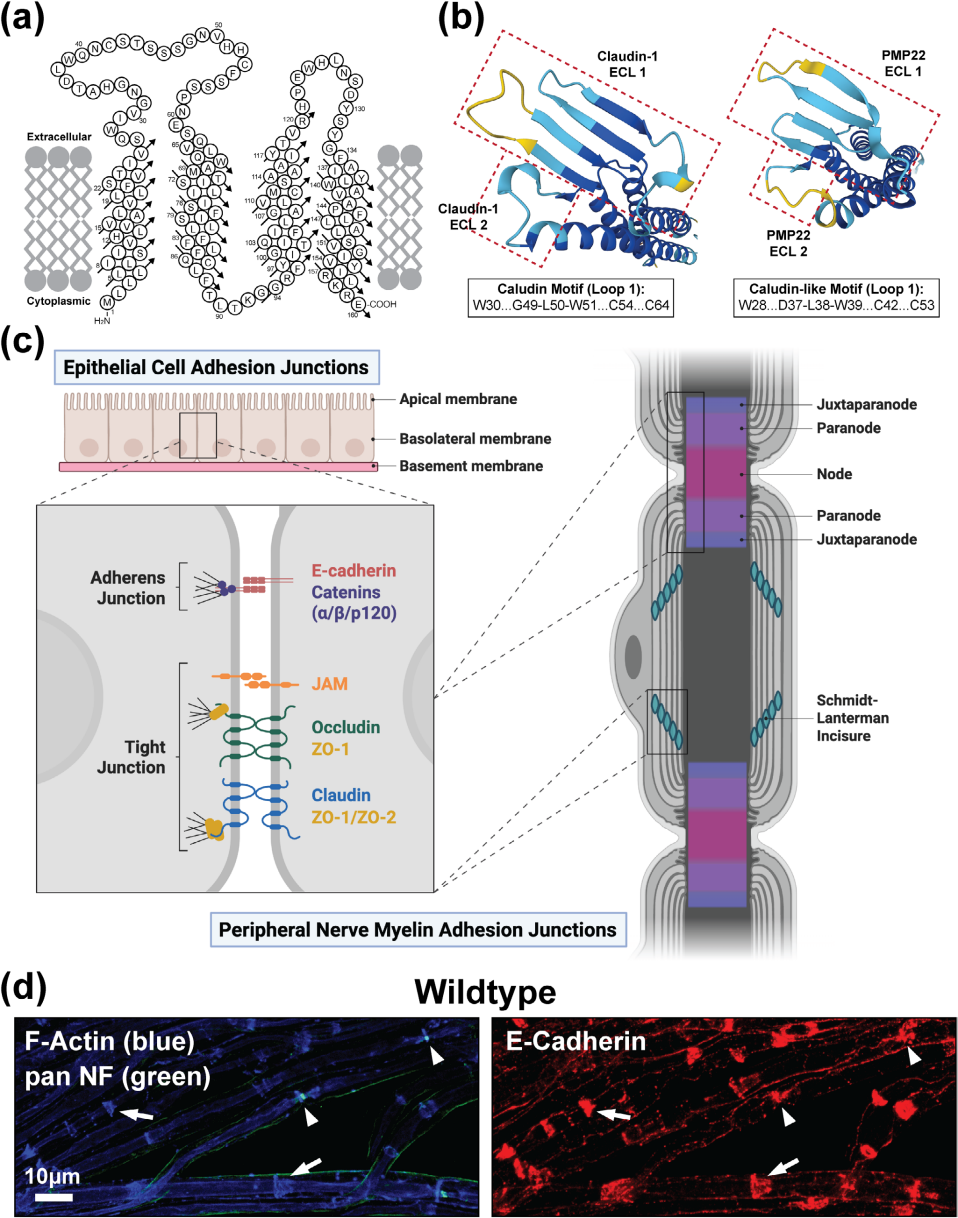
PMP22 exhibits structural homology to Claudins suggesting a role in myelin adhesion. (a) PMP22 is a member of the Claudin superfamily of proteins. Orthodox Claudins establish selective barriers as components of tight junctions and as such PMP22 protein topology is homologous. Topology for human PMP22, adapted from (Marinko et al. 2020). (b) AlphaFold predicted structures of human Claudin‐1 and human PMP22 ECLs viewed from above (AlphaFold Protein Structure Database (Jumper et al. 2021)). The structural similarity between the ECLs of human Claudin‐1 and human PMP22 is striking suggesting that PMP22 functions similarly to Claudin proteins. (c) Adhesion junctions, including adherens junctions and tight junctions, are localized to specialized cytoplasmic‐like compartments in peripheral nerve myelin including Schmidt‐Lanterman Incisures (SLIs) and Nodes of Ranvier. Created in https://BioRender.com. (d) 3‐month‐old WT (C57BL/6J) teased tibial nerve fibers immunostained for the adherens junction protein E‐Cadherin (red) showing prominent localization at SLIs (marked by F‐Actin [blue], arrows), Nodes of Ranvier (marked by pan Neurofascin [NF, green], arrowheads) and inner mesaxons. Scale bar, 10 μm.

PMP22 belongs to the Claudin superfamily, a group of transmembrane proteins best known for forming tight junctions that regulate paracellular permeability and maintain selective barriers (Simske 2013; Van Itallie and Anderson 2006). Consistent with this, PMP22 shares a homologous protein topology and its ECLs exhibit striking structural similarity to orthodox Claudins, like Claudin‐1, in both humans and mice (Figure 1a,b and Figure S1a). In contrast, ClustalW sequence alignments reveal only modest sequence similarity between PMP22 and Claudin‐1, despite strong conservation of PMP22 across species (Figure S1b). These findings reinforce the notion that PMP22 may carry out Claudin‐like functions through conserved structural features rather than high primary sequence identity. This role is likely critical for maintaining peripheral nerve myelin integrity, as adhesion junctions including tight and adherens junctions localize to specialized cytoplasmic‐like domains including SLIs and Nodes of Ranvier, where they are thought to play essential roles in myelin structural integrity and function (Fannon et al. 1995; Tricaud et al. 2005; Young et al. 2002) (Figure 1c).

### 3.2 Altered Adherens Junction Organization at SLIs in CMT1A and HNPP

**FIGURE 2:**
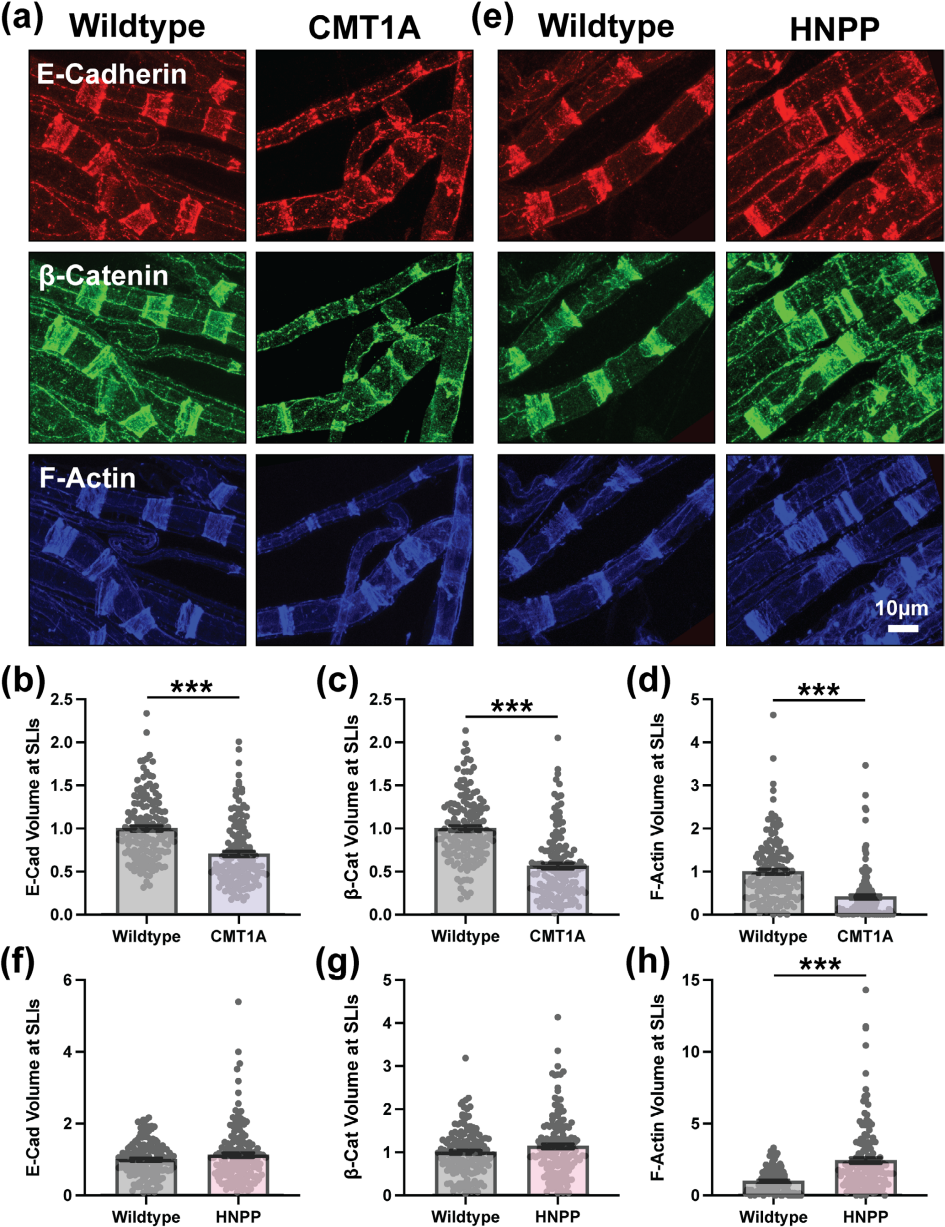
Altered adherens junctions and F‐actin distributions at SLIs in CMT1A and HNPP model peripheral nerve myelin. Representative images of 3‐month‐old (a) WT (C57BL/6J) and CMT1A or (e) WT (129S1/SvImJ) and HNPP teased tibial nerve fibers stained for the adherens junction complex proteins E‐Cadherin (red), β‐Catenin (green) and F‐Actin (blue). Quantification of protein distribution at SLIs (signal volume/fiber diameter) for (b, f) E‐Cadherin, (c, g) β‐Catenin and (d, h) F‐Actin in CMT1A and HNPP, respectively. *n* = 5 animals (~30 SLIs/animal). Bar graphs represent mean ± SEM with individual data points. Datasets were analyzed with three complementary statistical approaches (unpaired *t*‐test with all individual data points, unpaired *t*‐test with experimental means and nested *t*‐test). ***p < 0.05 demonstrate statistical significance with all three *t*‐test statistics. Scale bar, 10 μm.

To investigate the potential role of PMP22 in adhesion, we examined peripheral nerve myelin from constitutive mouse models of CMT1A (C3‐PMP) (Verhamme et al. 2011) and HNPP (PMP22‐deficient LacZ) (Adlkofer et al. 1995). Teased nerve fibers were prepared from adult mice (aged 3 months) by dissecting sciatic nerves, isolating the tibial branches and performing confocal immunofluorescence imaging. The tight junction component ZO‐1 demonstrated enrichment near Nodes of Ranvier (data not shown) and the adherens junction component E‐Cadherin was prominently enriched at SLIs, inner mesaxons and at Nodes of Ranvier (Figure 1d). CMT1A fibers did not show marked changes in ZO‐1 (data not shown); however, E‐Cadherin was severely disrupted, with striking disorganization particularly evident at SLIs (Figure 2a and Figure S2a). The signal was more punctate and the funnel shape structure of SLIs was often disrupted. Quantification of this defect revealed a decrease in E‐Cadherin signal volume normalized to fiber diameter (hereafter referred to as *distribution*). This metric accounts for the shift from large‐ to small‐caliber fibers in CMT1A model mice (Moss et al. 2022; Verhamme et al. 2011) and captures changes in spatial patterning within myelin. All datasets throughout were analyzed using three complementary statistical approaches, arranged from least to most stringent: (1) unpaired *t*‐test with all individual data points (each SLI, single asterisk), (2) unpaired *t*‐test with experimental means (*n* = 5, double asterisk), and (3) nested *t*‐test comparing individual data points grouped by experiment (triple asterisk). Significant fold changes are reported alongside the most stringent *p*‐value. E‐Cadherin at SLIs was compacted as demonstrated by reduced distribution in CMT1A model mice (Figure 2b; 0.704‐fold change, nested *t*‐test *p* = 0.0118). Mean E‐Cadherin signal intensity at SLIs was modestly increased reaching statistical significance but is likely not biologically significant due to the small fold change (Figure S2b; 1.098‐fold change, unpaired *t*‐test with individual data points *p* = 0.0007). The core complex of adherens junctions consists of the single pass transmembrane protein E‐cadherin and three cytoplasmic Catenin proteins (p120‐Catenin, β‐Catenin, and α‐Catenin) which connect the cadherin complex to the actin cytoskeleton and multiple signaling pathway effectors (Campas et al. 2024; Troyanovsky 2023). The distributions of β‐Catenin and F‐Actin mimicked the changes in E‐Cadherin but exhibited even stronger changes (Figure 2a,c,d; β‐Catenin: 0.567‐fold change, nested *t*‐test *p* = 0.0028; F‐Actin: 0.414‐fold change, nested *t*‐test *p* = 0.0024). Whereas mean β‐Catenin and F‐Actin signal intensity at SLIs was unchanged in CMT1A (Figure 2a and Figure S2a,c,d). These findings suggest that adherens junctions at SLIs and likely the overall morphology of SLIs, as evidenced by dramatic changes in F‐Actin, are compromised in myelin from CMT1A model mice exhibiting an abnormally compacted distribution.

**FIGURE 3:**
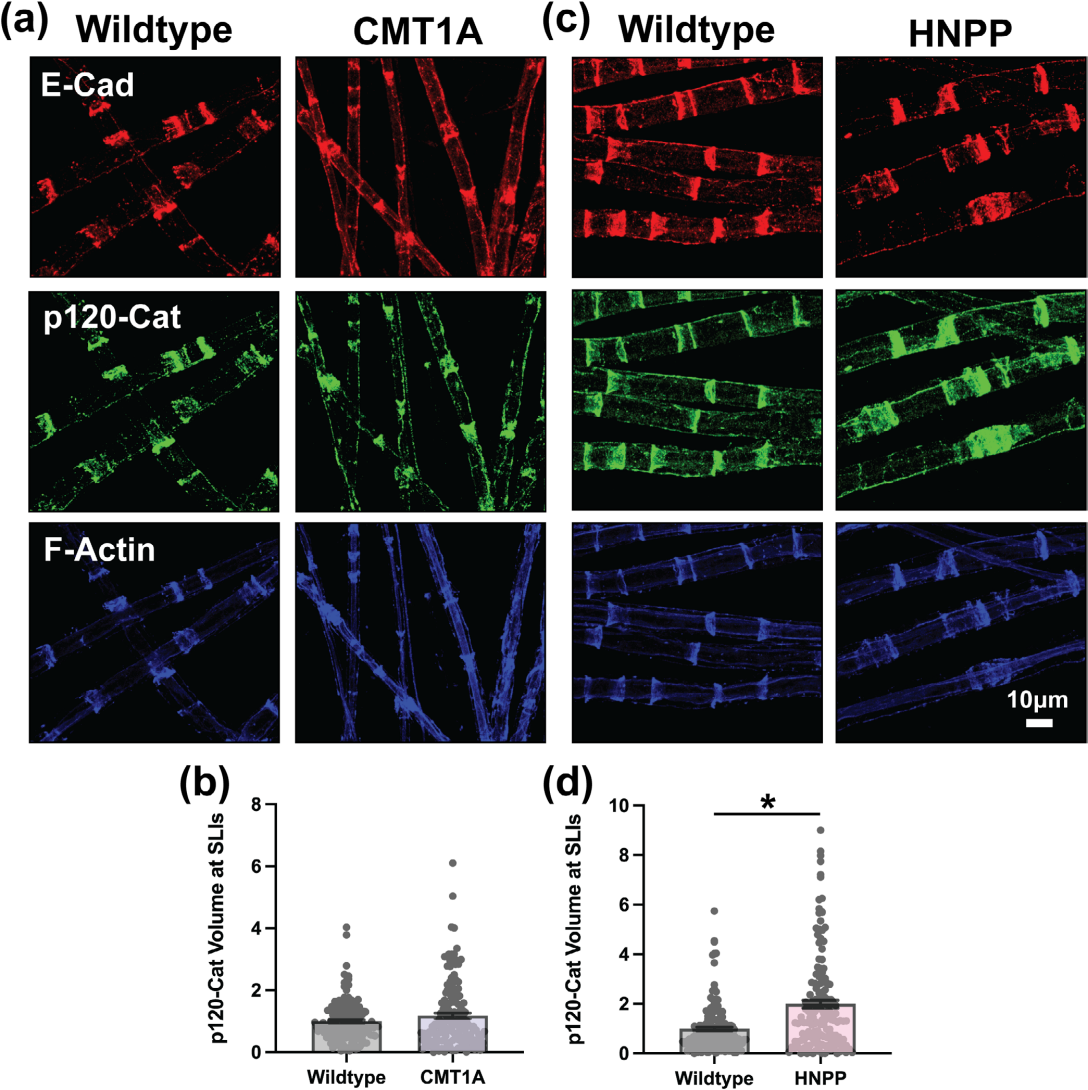
Variable changes in the distribution of p120‐Catenin at SLIs in CMT1A and HNPP model myelin. Representative images of 3‐month‐old (a) WT (C57BL/6J) and CMT1A or (c) WT (129S1/SvImJ) and HNPP teased tibial nerve fibers stained for the adherens junction complex proteins E‐Cadherin (red), p120‐Catenin (green) and F‐Actin (blue). Quantification of p120‐Catenin protein distribution at SLIs (signal volume/fiber diameter) in (b) CMT1A and (d) HNPP. *n* = 5 animals (~30 SLIs/animal). Bar graphs represent mean ± SEM with individual data points. Datasets were analyzed with three complementary statistical approaches (unpaired *t*‐test with all individual data points, unpaired *t*‐test with experimental means and nested *t*‐test). *p < 0.05 demonstrate statistical significance with a single *t*‐test statistic. Scale bar, 10 μm.

In peripheral nerve myelin from HNPP model mice, E‐Cadherin and β‐Catenin remained largely unchanged, with only mean β‐Catenin signal intensity at SLIs reaching statistical significance (1.106‐fold change, unpaired *t*‐test with individual data points *p* = 0.0033) but likely lacking biological relevance (Figure 2e–g and Figure S2e–g). However, overall SLI morphology is likely oppositely affected in HNPP as evidenced by increased distribution and increased mean signal intensity of F‐Actin (Figure 2e,h and Figure S2e,h; Distribution: 2.468‐fold change, nested *t*‐test *p* = 0.0044; Mean: 1.352‐fold change, nested *t*‐test *p* = 0.0316). β‐Catenin and F‐Actin are key components of adherens junctions, where they contribute to both structural stability and dynamic regulation of cell adhesion. β‐Catenin binds the intracellular domain of E‐Cadherin and associates with α‐Catenin, which in turn links to F‐Actin (Campas et al. 2024; Troyanovsky 2023). Together, these interactions bridge adherens junctions with the actin cytoskeleton, supporting adherens junction integrity. In peripheral nerve, α3‐Catenin is reported to be the predominant isoform for which reliable antibodies are limited, preventing its inclusion in this study (Weng et al. 2022). The third catenin in the core adherens junction complex, p120‐Catenin, binds the intracellular domain of E‐Cadherin, where it regulates E‐Cadherin stability by preventing endocytosis and degradation and modulates small GTPase signaling to influence actin dynamics and junctional organization (Jin et al. 2024; Kourtidis et al. 2013). p120‐Catenin distribution at SLIs was unchanged in myelin from CMT1A model mice but was significantly increased in HNPP (Figure 3a–d; 2.002‐fold change, unpaired *t*‐test with individual data points *p* < 0.0001). However, p120‐Catenin mean signal intensity at SLIs was modestly increased in both CMT1A (1.091‐fold change, unpaired *t*‐test with individual data points *p* = 0.0400) and HNPP (1.112‐fold change, unpaired *t*‐test with individual data points *p* = 0.0033) although unlikely biologically significant (Figure 3a,c and Figure S3a–d). Given that p120‐Catenin stabilizes E‐Cadherin at the membrane (Jin et al. 2024; Kourtidis et al. 2013), the increased distribution of p120‐Catenin at SLIs may represent a compensatory response to adherens junction disruption, potentially resulting in better preservation of junctional integrity in HNPP than CMT1A.

### 3.3 Additional SLI Abnormalities With Divergent Features in CMT1A and HNPP

**FIGURE 4:**
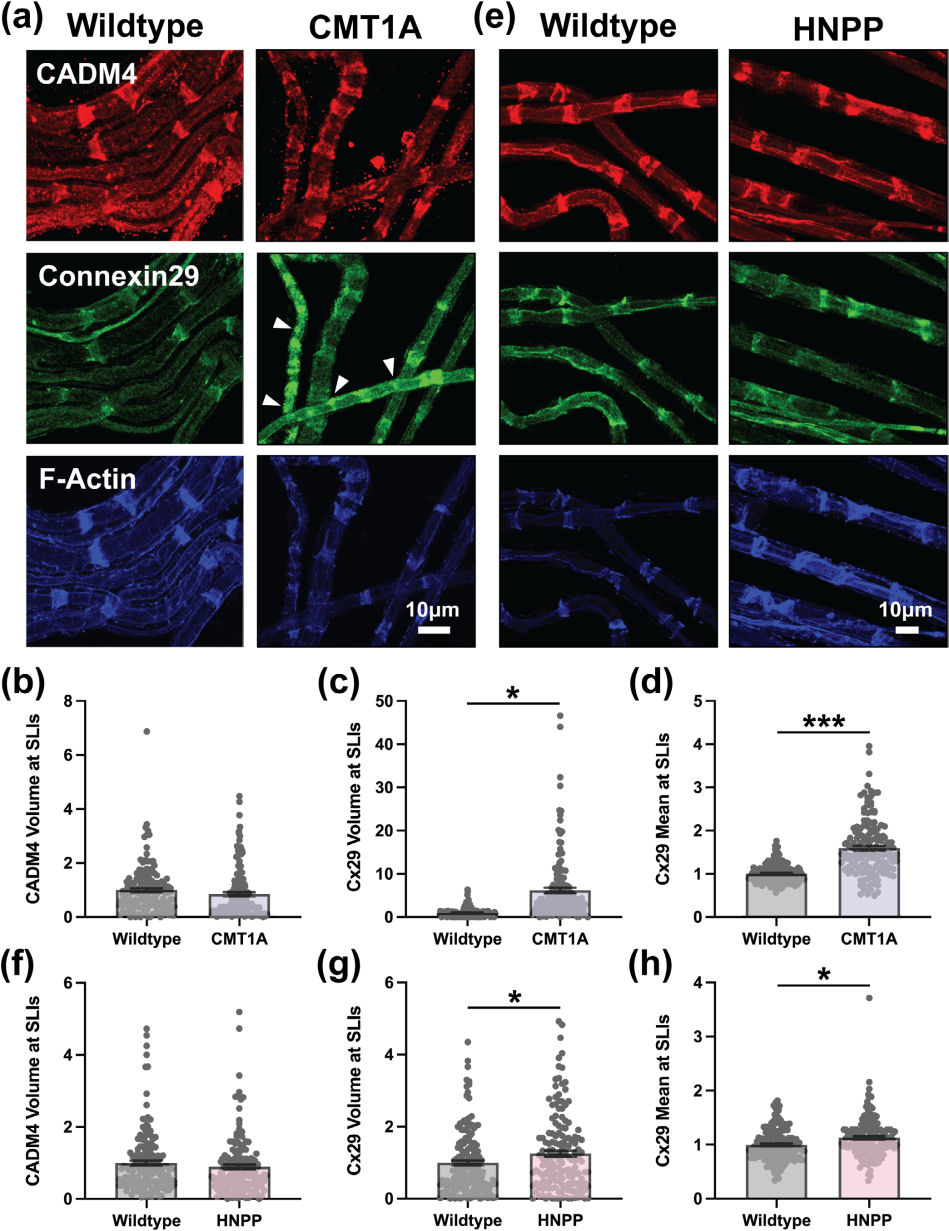
Distinct Connexin29 distribution defects but unchanged CADM4 at SLIs in peripheral nerve myelin from CMT1A and HNPP model mice. Representative images of 3‐month‐old (a) WT (C57BL/6J) and CMT1A or (e) WT (129S1/SvImJ) and HNPP teased tibial nerve fibers stained for CADM4 (red), Connexin29 (Cx29, green) and F‐Actin (blue). Note the focal accumulations of Cx29 outside the SLI compartment in CMT1A model myelin (arrowheads). Quantification of protein distribution at SLIs (signal volume/fiber diameter) for (b, f) CADM4 and (c, g) Cx29 in CMT1A and HNPP, respectively. Quantification of mean Cx29 signal intensity at SLIs in (d) CMT1A and (h) HNPP. *n* = 5 animals (~30 SLIs/animal). Bar graphs represent mean ± SEM with individual data points. Datasets were analyzed with three complementary statistical approaches (unpaired *t*‐test with all individual data points, unpaired *t*‐test with experimental means and nested *t*‐test). ***p < 0.05 demonstrate statistical significance with all three *t*‐test statistics and *p < 0.05 demonstrate statistical significance with a single *t*‐test statistic. Scale bars, 10 μm.

Given our prediction that overall SLI morphology is disrupted in CMT1A and HNPP model myelin, we next examined whether other SLI components displayed similar alterations to those observed for adherens junction proteins and F‐Actin. Cell Adhesion Molecule 4 (CADM4 but also known as SynCAM4 or Necl‐4), Connexin29 (Cx29), and MAG are key molecular components of peripheral nerve myelin that are enriched at SLIs and contribute to axoglial communication and structural organization (Rash et al. 2016; Altevogt et al. 2002; Meng et al. 2019; Schnaar and Lopez 2009). Additionally, Connexin32 (Cx32) is a gap junction protein enriched at SLIs and essential for myelin integrity, as mutations in the gene encoding this protein (*GJB1*) cause CMT Type 1X (CMT1X) (Scherer et al. 1998; Balice‐Gordon et al. 1998; Bergoffen et al. 1993). CADM4 mean signal intensity and distribution at SLIs were unchanged in both CMT1A and HNPP indicating that not all SLI components are altered by these measures (Figure 4a,b,e,f and Figure S4a–d). However, Cx29 and MAG show striking and similar alterations in their distributions and mean signal intensity at SLIs in CMT1A and HNPP, differing from the changes observed for adherens junctions and F‐Actin. Cx29 distribution was dramatically increased at SLIs in CMT1A (Figure 4a,c and Figure S4a; 6.233‐fold change, unpaired *t*‐test with individual data points *p* < 0.0001) and more modestly in HNPP (Figure 4e,g and Figure S4c; 1.257‐fold change, unpaired *t*‐test with individual data points *p* = 0.0205). Similarly, Cx29 mean signal intensity at SLIs was increased more so in CMT1A (Figure 4a,d and Figure S4a; 1.596‐fold change, nested *t*‐test *p* = 0.0162) than HNPP (Figure 4e,h and Figure S4c; 1.132‐fold change, unpaired *t*‐test with individual data points *p* = 0.0205).

**FIGURE 5:**
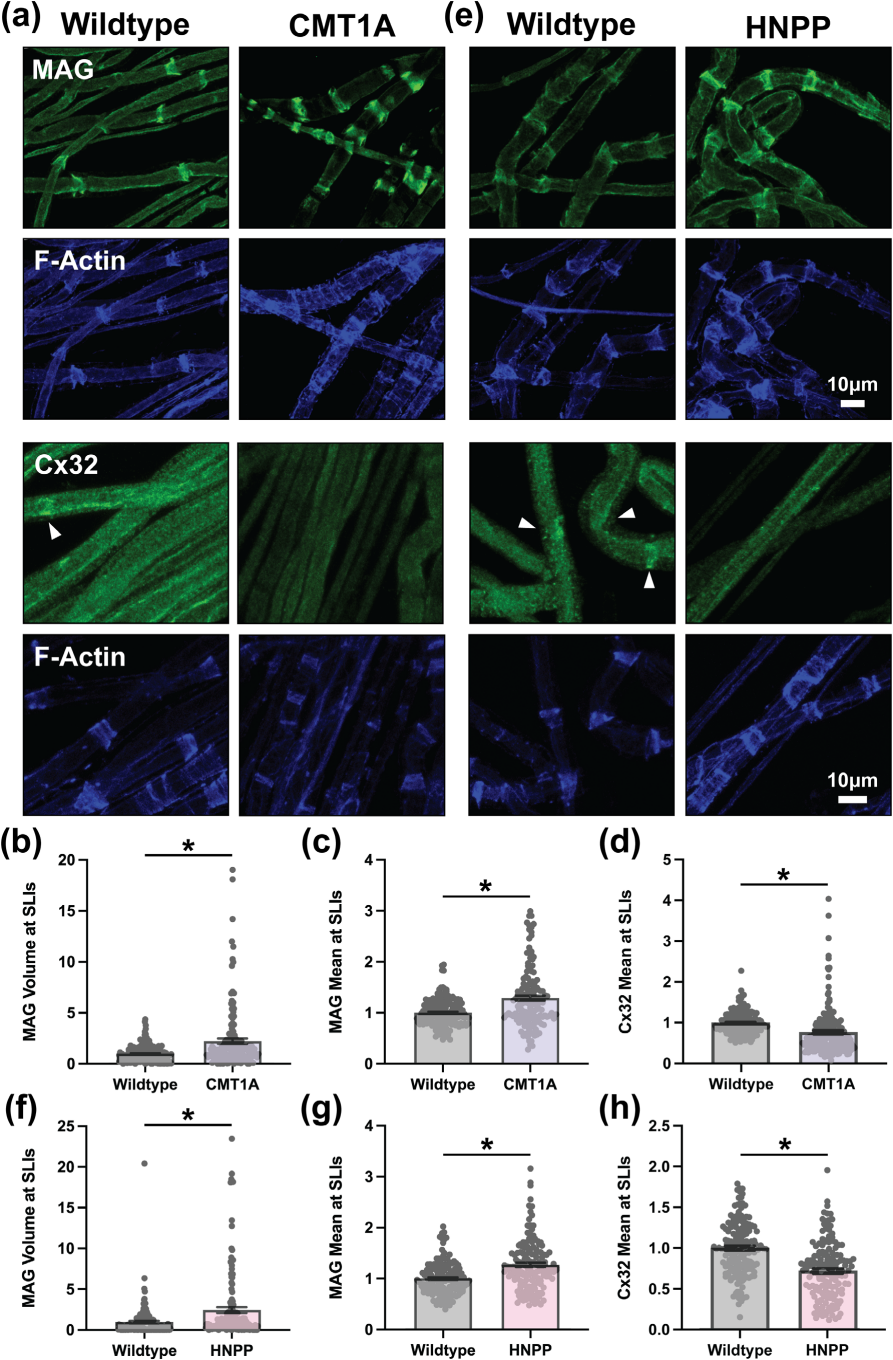
Mag exhibits distribution changes similar to Connexin29 whereas Connexin32 mean intensity is reduced at SLIs in CMT1A and HNPP model myelin. Representative images of 3‐month‐old (a) WT (C57BL/6J) and CMT1A or (e) WT (129S1/SvImJ) and HNPP teased tibial nerve fibers stained for *(top panels)* MAG (green) and F‐Actin (blue) or *(bottom panels)* Cx32 (green) and F‐Actin (blue). Quantification of (b, f) MAG protein distribution (signal volume/fiber diameter) and (c, f) MAG and (d, h) Cx32 mean signal intensity at SLIs in CMT1A and HNPP, respectively. *n* = 5 animals (~30 SLIs/animal). Bar graphs represent mean ± SEM with individual data points. Datasets were analyzed with three complementary statistical approaches (unpaired *t*‐test with all individual data points, unpaired *t*‐test with experimental means and nested *t*‐test). *p < 0.05 demonstrate statistical significance with a single *t*‐test statistic. Scale bars, 10 μm.

MAG distribution at SLIs was increased in CMT1A (2.232‐fold change, unpaired *t*‐test with individual data points *p* < 0.0001) and HNPP (2.471‐fold change, unpaired *t*‐test with individual data points *p* = 0.0002) to a similar degree (Figure 5a,b,e,f and Figure S5a,b). MAG mean signal intensity at SLIs was also comparably increased in CMT1A (Figure 5a,c; 1.290‐fold change, unpaired *t*‐test with individual data points *p* < 0.0001) and HNPP (Figure 5e,g; 1.270‐fold change, unpaired *t*‐test with individual data points *p* < 0.0001). Cx32 exhibited variable enrichment at SLIs precluding reliable distribution analysis. However, mean signal intensity of Cx32 at SLIs was reduced in both CMT1A (Figure 5a,d and Figure S5a; 0.7745‐fold change, unpaired *t*‐test with individual data points *p* < 0.0001) and HNPP (Figure 5e,h and Figure S5b; 0.7197‐fold change, unpaired *t*‐test with individual data points *p* < 0.0001). These results collectively reveal that not all SLI components are equally affected in CMT and HNPP model mice. Particularly notable are those that show opposite effects in CMT1A and HNPP, reflecting the inverse changes in *PMP22* expression, such as F‐Actin distribution. Others, like Cx29 distribution and mean signal intensity, that are more severely disrupted in CMT1A than in HNPP are consistent with the relative phenotype severity of the corresponding mouse models and potentially indicative of a shared pathomechanistic basis in these models. Cx29 was also frequently observed in focal accumulations along the internode outside of SLIs in CMT1A (Figure 4a and Figure S4a) and sometimes in HNPP, suggesting that additional mechanisms beyond SLI‐specific disruptions may occur in these models. Given that Cx29 and other SLI‐associated proteins also localize to the inner mesaxon and to Nodes of Ranvier (Scherer and Arroyo 2002), we turned our attention to nodal architecture, both due to its critical role in action potential propagation and because nodopathy is a well‐established mechanism in acquired neuropathies.

### 3.4 Peripheral Nerve Ion Channel Deficits at Nodes of Ranvier in CMT1A and HNPP

**FIGURE 6:**
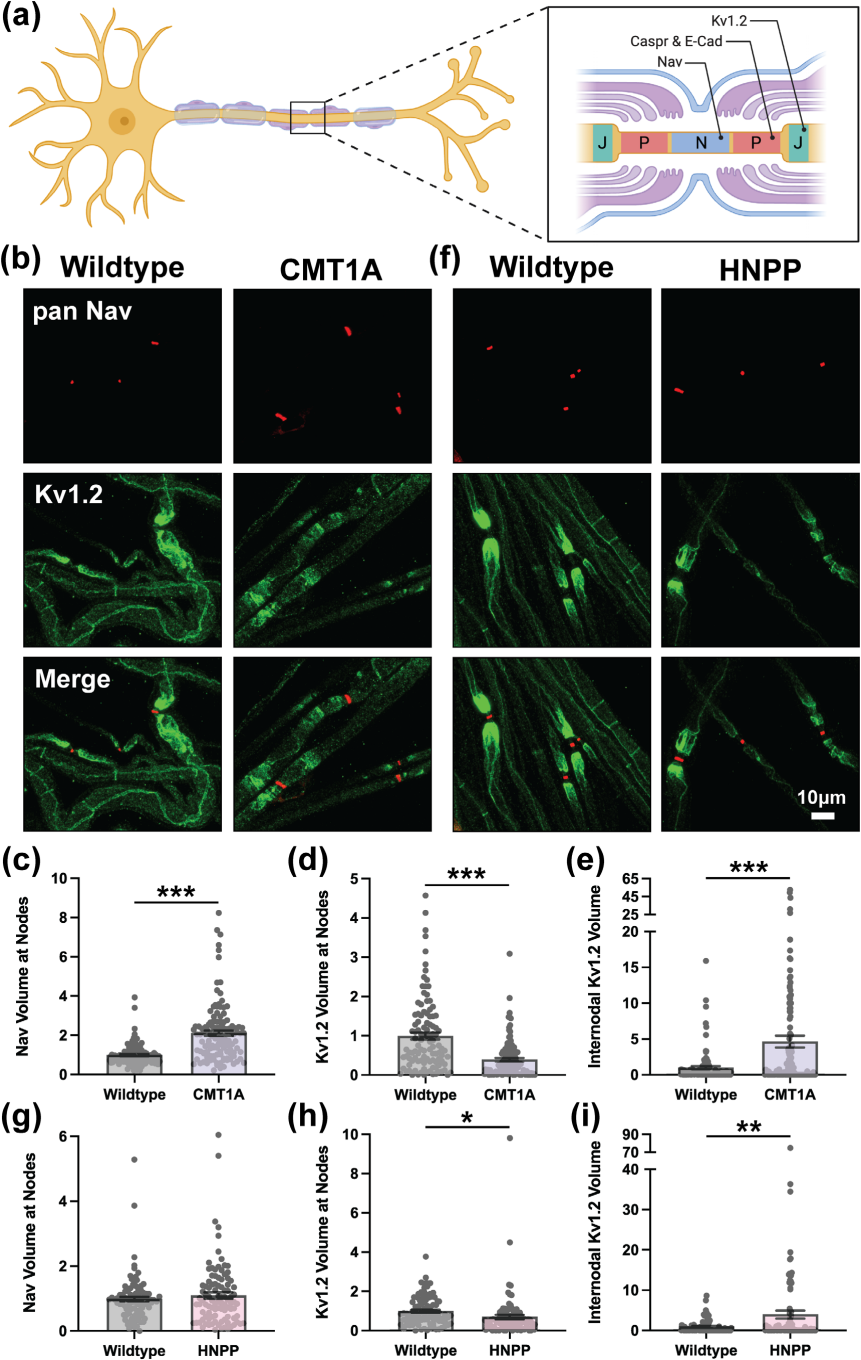
Disrupted ion channel distribution at nodes of Ranvier in CMT1A and HNPP model peripheral nerve myelin. (a) Diagram demonstrating the molecular organization of three distinct subdomains at Nodes of Ranvier: Nodes (N, containing Nav), paranodes (P, containing Caspr and E‐Cadherin) and Juxtaparanodes (J, containing Kv1.2). Created in https://BioRender.com. Representative images of 3‐month‐old (b) WT (C57BL/6J) and CMT1A or (f) WT (129S1/SvImJ) and HNPP teased tibial nerve fibers stained with anti‐Pan Nav (red) and anti‐Kv1.2 (green). Quantification of protein distribution at Nodes of Ranvier (signal volume/fiber diameter) for (c, g) Nav and (d, h) Kv1.2 in CMT1A and HNPP, respectively. (e, i) Quantification Kv1.2 internodal spread away from Nodes of Ranvier in CMT1A and HNPP, respectively. *n* = 5 animals (~15–25 nodes/animal). Bar graphs represent mean ± SEM with individual data points. Datasets were analyzed with three complementary statistical approaches (unpaired *t*‐test with all individual data points, unpaired *t*‐test with experimental means and nested *t*‐test). ***p < 0.05 demonstrate statistical significance with all three *t*‐test statistics, **p < 0.05 demonstrate statistical significance with two *t*‐test statistics and *p < 0.05 demonstrate statistical significance with a single *t*‐test statistic. Scale bar, 10 μm.

Nodes of Ranvier are spatially organized into three distinct subdomains, the node, paranode and juxtaparanode, to allow for saltatory conduction of action potentials along axons (Figure 6a). The node contains voltage‐gated sodium channels critical for depolarization, with Nav1.6 predominating at mature nodes that support high‐frequency peripheral nerve axon firing, while Nav1.7, Nav1.8, and Nav1.9 are primarily enriched at nodes in sensory neurons (Bao 2015; Caldwell et al. 2000). The juxtaparanode contains voltage‐gated potassium channels essential for repolarization, with Kv1.1 and Kv1.2 as the predominant subtypes in fast‐conducting peripheral nerve axons (Rash et al. 2016). The paranode contains septate‐like junctions formed by axonal Caspr and Contactin and Schwann cell Neurofascin 155, which anchor the terminal loops of each myelin wrap to the axon and functionally separate the node from the juxtaparanode (Faivre‐Sarrailh 2020). Further motivation to investigate Node of Ranvier architecture arises from findings that Cx29 co‐localizes with Kv1 channels at juxtaparanodes, suggesting electrical coupling between axons and myelinating Schwann cells, and that adherens junctions localize to paranodes where they are proposed to contribute to paranodal integrity (Rash et al. 2016; Fannon et al. 1995). Nodes and juxtaparanodes were assessed in teased nerve fibers from adult CMT1A and HNPP model mice by staining with anti‐pan Nav and anti‐Kv1.2 antibodies, respectively. Nav distribution at nodes was increased in CMT1A (2.122‐fold change, nested *t*‐test *p* = 0.0125) but unchanged in HNPP (Figure 6b,c,f,g and Figure S6a,d). Additionally, Nav mean signal intensity at nodes was increased in CMT1A (1.566‐fold change, unpaired *t*‐test with individual data points *p* < 0.0001) but unchanged in HNPP (Figure 6b,f and Figure S5a,b,d,e). Together, these results suggest that nodal widening, a well‐documented feature of peripheral nerve pathology (Hildebrand 1989; Arroyo et al. 1999), is prominent in CMT1A model myelin but not in HNPP.

We also observed pronounced alterations in Kv1.2 distribution in myelin from CMT1A and HNPP model mice. Kv1.2 distribution near Nodes of Ranvier was reduced, with a more pronounced reduction in CMT1A (Figure 6b,d and Figure S6a; 0.4026‐fold, nested *t*‐test *p* = 0.0003) as compared to HNPP (Figure 6f,h and Figure S6d; 0.7019‐fold, unpaired *t*‐test with individual data points *p* = 0.0305). Kv1.2 mean signal intensity near Nodes of Ranvier was also reduced in CMT1A and HNPP but to a similar degree (CMT1A: Figure 6b and Figure S6a,c; 0.7968‐fold change, unpaired *t*‐test with individual data points *p* < 0.0001 and HNPP: Figure 6f and Figure S6d,f; 0.7916‐fold change, unpaired *t*‐test with individual data points *p* < 0.0001). In addition, Kv1.2 was frequently found to be spread along the internode away from the juxtaparanodal compartment. Quantification of this was performed by measuring total Kv1.2 signal volume across the Node of Ranvier and proximal internode, subtracting the juxtaparanodal contribution, and normalizing the result to the total volume of the selected fiber segment. Internodal Kv1.2 distribution was markedly increased in both CMT1A and HNPP model myelin, with a more pronounced elevation in CMT1A (CMT1A: Figure 6b,e; 4.716‐fold change, nested *t*‐test *p* = 0.0188 and HNPP: Figure 6f,i; 3.987‐fold change, unpaired *t*‐test with experimental means *p* = 0.0402). These pronounced changes at Nodes of Ranvier, including nodal widening and altered Kv1.2 localization, may compromise nodal architecture and contribute to impaired conduction, with greater disruption evident in CMT1A than in HNPP.

### 3.5 Compromised Paranode Integrity at Nodes of Ranvier in CMT1A and HNPP

**FIGURE 7:**
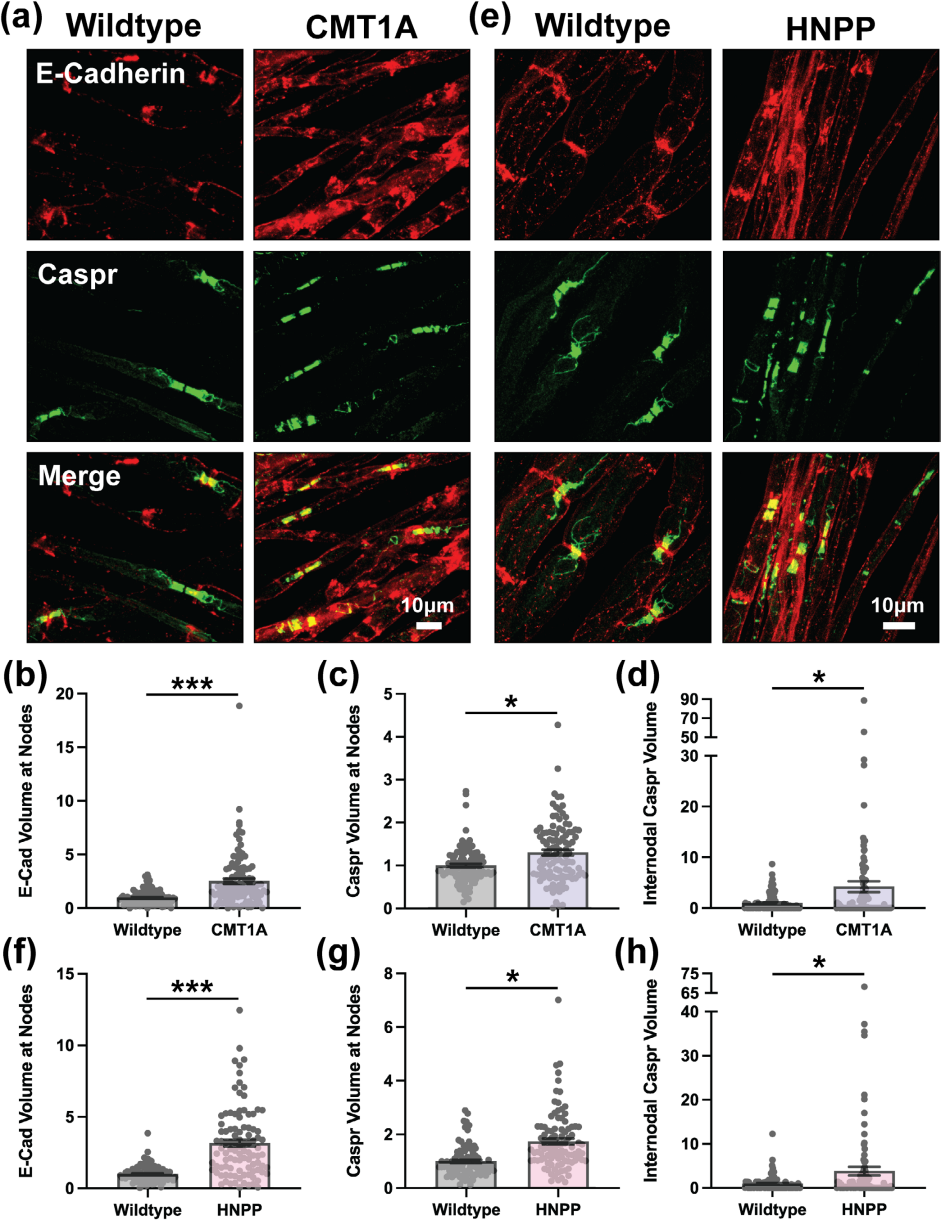
Altered Caspr and E‐Cadherin distributions at paranodes CMT1A and HNPP model myelin. Representative images of 3‐month‐old (a) WT (C57BL/6J) and CMT1A or (e) WT (129S1/SvImJ) and HNPP teased tibial nerve fibers stained for E‐Cadherin (red) and Caspr (green). Quantification of protein distribution at Nodes of Ranvier (signal volume/fiber diameter) for (b, f) E‐Cadherin and (c, g) Caspr in CMT1A and HNPP, respectively. (d, h) Quantification of Caspr internodal spread away from Nodes of Ranvier in CMT1A and HNPP, respectively. *n* = 5 animals (~15–25 nodes/animal). Bar graphs represent mean ± SEM with individual data points. Datasets were analyzed with three complementary statistical approaches (unpaired *t*‐test with all individual data points, unpaired *t*‐test with experimental means and nested *t*‐test). ***p < 0.05 demonstrate statistical significance with all three *t*‐test statistics and *p < 0.05 demonstrate statistical significance with a single *t*‐test statistic. Scale bars, 10 μm.

Given the essential role of paranodal septate‐like junctions in maintaining nodal organization (Rasband and Peles 2025), we next evaluated whether disruptions in this domain align with the nodal changes observed in CMT1A and HNPP model mice. We evaluated a key component of paranodes, Caspr (also called Caspr1), by confocal immunofluorescence imaging. Caspr plaques at the paranode were often fragmented and focal accumulations of Caspr were often visible along the internode like the distribution of Kv1.2. However, these abnormalities were comparable in magnitude between CMT1A and HNPP. Caspr distribution near Nodes of Ranvier was increased (CMT1A: Figure 7a,c and Figure S7a; 1.305‐fold change, unpaired *t*‐test with individual data points *p* = 0.0002 and HNPP: Figure 7e,g and Figure S7d; 1.739‐fold change, unpaired *t*‐test with individual data points *p* < 0.0001) and Caspr mean signal intensity near Nodes of Ranvier was modestly increased (CMT1A: Figure 7a and Figure S7a,c; 1.106‐fold change, unpaired *t*‐test with individual data points *p* = 0.0129 and HNPP: Figure 7e and Figure S7d,f; 1.255‐fold change, unpaired *t*‐test with individual data points *p* < 0.0001). Internodal Caspr distribution was quantified using the same method as for Kv1.2, revealing a dramatic increase in both CMT1A and HNPP, potentially to a greater extent in CMT1A (CMT1A: Figure 7a,d; 4.263‐fold change, unpaired *t*‐test with individual data points *p* = 0.0034 and HNPP: Figure 7e,h; 3.871‐fold change, unpaired *t*‐test with individual data points *p* = 0.0046).

E‐Cadherin at Nodes of Ranvier was also examined, given the striking alterations in adherens junction components at SLIs in CMT1A model myelin. E‐Cadherin distribution near Nodes of Ranvier was increased in both CMT1A and HNPP, potentially to a greater extent in HNPP (CMT1A: Figure 7a,b and Figure S7a; 2.623‐fold change, nested *t*‐test *p* = 0.0335 and HNPP: Figure 7e,f and Figure S6d; 3.118‐fold change, nested *t*‐test *p* = 0.0138), while mean signal intensity was similarly increased in both models (CMT1A: Figure 7a and Figure S7a,b; 1.324‐fold change, nested *t*‐test *p* = 0.0061 and HNPP: Figure 7e and Figure S7d,e; 1.338‐fold change, nested *t*‐test *p* = 0.0239). The more pronounced changes in Caspr and E‐Cadherin distributions and mean signal intensities at Nodes of Ranvier observed in HNPP as compared to CMT1A do not correlate with the relative phenotype severity of the corresponding mouse models. These findings are potentially influenced by the presence of tomacula, focal myelin thickenings that frequently occur near the node in HNPP (Adlkofer et al. 1995), complicating interpretation. This confounds the conclusions drawn from these experiments but lends greater weight to deficits more severely affected in CMT1A than in HNPP as potential drivers of disease pathogenesis.

### 3.6 Myelin Structural Abnormalities Occur in CMT1A and HNPP Fibers With Preserved Compact Myelin and Arise During Development

To determine whether the molecular alterations observed in CMT1A and HNPP model myelin could be secondary to overt demyelination or to the presence of degenerating and regenerating myelin sheaths, we carefully examined overall myelin morphology of the fibers analyzed in our datasets. Segmental demyelination in HNPP model mice has been reported to emerge only after approximately 12–15 months of age (Adlkofer et al. 1997), whereas this feature has not been definitively described in CMT1A models. However, several studies have noted the presence of amyelinated fibers in the C3‐PMP mouse model of CMT1A using toluidine blue staining and electron microscopy cross‐sectional analysis. Reported frequencies vary across studies and nerve regions, including lower proportions in dorsal root (7.6%) (Hantke et al. 2014) and saphenous (0.5%) (Hantke et al. 2014) and sciatic nerves (5%–8%) (Moss et al. 2022), with higher frequencies in femoral nerve (~10%–27%) (Verhamme et al. 2011; Bai et al. 2022) and ventral root (47.9%) (Hantke et al. 2014). A related CMT1A mouse model, C61, also contains a small proportion of amyelinated fibers in sciatic nerve (~5%) (Robertson et al. 2002). Based on these prior findings, we did not anticipate observing abundant segmental demyelination in tibial branch teased fiber preparations from 3‐month‐old CMT1A and HNPP model mice. As expected, we did not detect prominent segmental demyelination during imaging and therefore did not exclude fibers containing heminodes from our analysis. Tiled widefield fluorescence microscopy of peripheral nerve fibers stained with anti‐βIII‐tubulin and anti‐pan Neurofascin antibodies, used to label axons and myelin respectively, further confirmed that segmental demyelination was not a prominent feature in the 3‐month‐old CMT1A and HNPP nerves we examined (Figure S8a). In addition, we previously reported that the average G‐Ratios remain unchanged in CMT1A model mice due to the presence of thickly myelinated small‐caliber fibers, which offset the amyelinated and thinly myelinated large‐caliber fibers (Moss et al. 2022). To confirm the myelinated fibers that were analyzed for molecular changes in the present study exhibited grossly normal myelin thickness, we measured axonal diameters in a subset of images using F‐actin staining and combined these with previously obtained total fiber diameters to calculate G‐Ratios (Figure S8b). Average G‐Ratios graphed with all individual data points (Figure S8c,e) and by experimental means (Figure S18d,f) were mostly unchanged. Only CMT1A average G‐Ratio with all individual data points reached statistical significance (WT Mean: 0.5228, CMT1A Mean: 0.5634, 1.078‐fold change, unpaired *t*‐test *p* = 0.0022) but likely lacking biological relevance due to the small fold change (Figure S8c and Figure S2e–g). Further support for grossly preserved myelin in the analyzed fibers is evident from G‐Ratio versus axon diameter plots, which demonstrate that G‐Ratios were more consistent across different axon calibers in WT mice, whereas CMT1A mice exhibited a stronger positive correlation between these variables, as we previously reported (Moss et al. 2022) (Figure S8g). In contrast, WT and HNPP mice displayed similar trendlines (Figure S8h). When the complete dataset is considered, the vast majority of fibers fall within the normal to thick myelin range (≤ 0.7) across all axon calibers, a pattern consistent among WT, CMT1A and HNPP model mice. Additionally, plots of G‐ratio versus nodal protein distribution (Nav, Kv1.2, and Caspr) demonstrate the lack a of strong correlation between these variables and show that fibers with the most severe nodal phenotypes do not consistently exhibit thinner myelin (Figure S9a–f). Together, these findings indicate that the CMT1A and HNPP fibers that we examined exhibit molecular alterations in the absence of overt demyelination.

To assess whether the molecular alterations observed in CMT1A and HNPP arise early during disease pathogenesis, we examined developing peripheral nerves at postnatal day 15 (P15). Teased nerve fibers were prepared from sciatic nerves by isolating tibial branches and analyzed by confocal immunofluorescence imaging. Both SLI (E‐Cadherin, β‐Catenin, F‐Actin, and MAG; Figure S10a,b) and Node of Ranvier (Nav and Caspr; Figure S10c,d) components were evaluated at this stage. At P15, myelination is still ongoing, but myelin domain patterning has already begun and can be reliably assessed (Chen et al. 2024; Garbay et al. 2000). All SLI components exhibited altered distributions in CMT1A mice, consistent with the changes observed in adults, whereas HNPP mice showed potentially more pronounced alterations in SLI components at this early stage. Likewise, nodal abnormalities at P15 mirrored the adult phenotypes in both CMT1A and HNPP, including fragmented and internodally displaced Caspr staining and nodal widening of Nav, which was qualitatively more severe in CMT1A.

**FIGURE 8:**
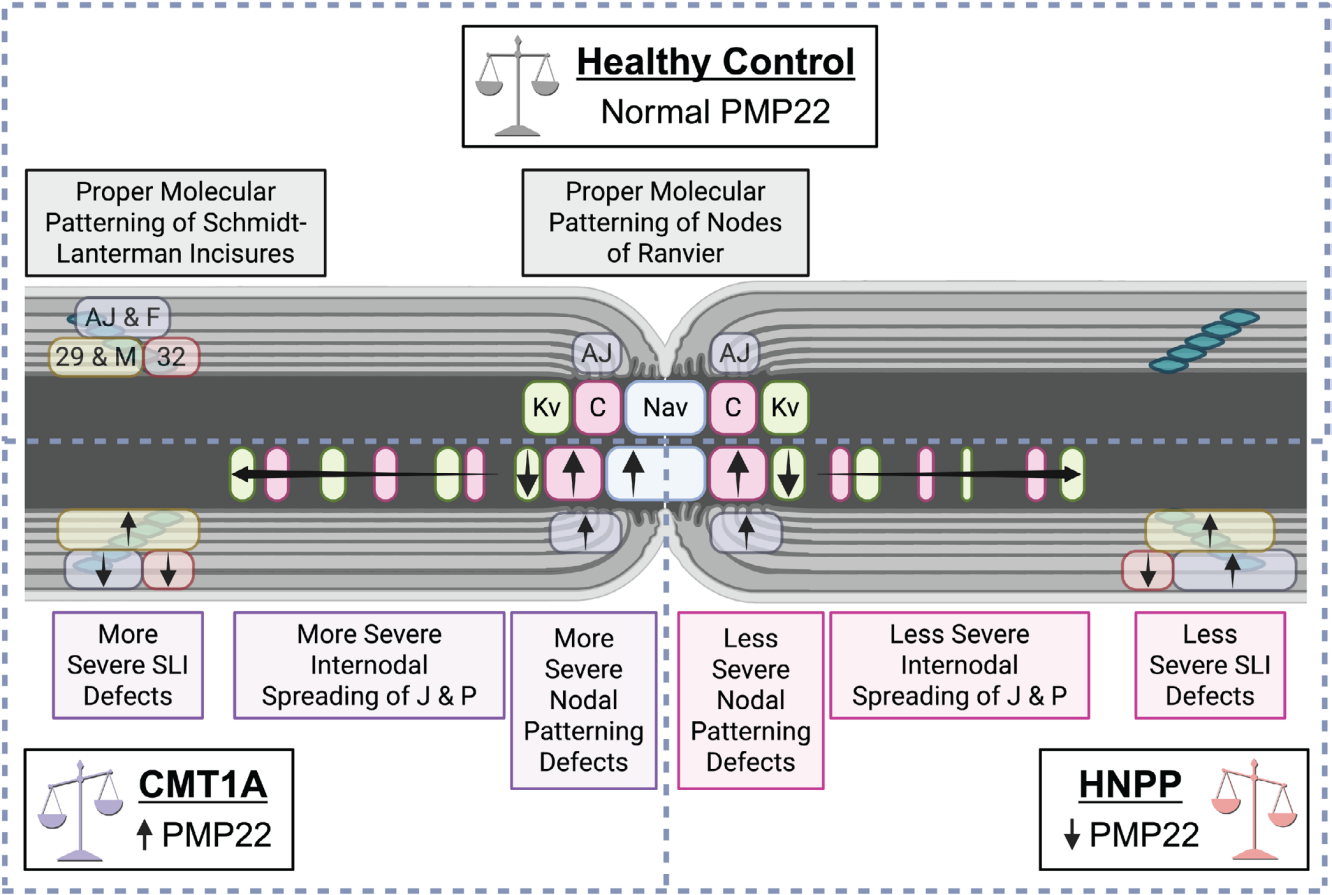
Overview of molecular alterations in relation to mouse model disease severity. Because CMT1A is typically a more severe disease than HNPP, with earlier onset and greater functional impairment, molecular and cellular phenotypes that are more pronounced in CMT1A, or that display opposing trends between the two models in accordance with *PMP22* gene dosage, are likely to align with disease burden and represent key contributors to pathogenesis. Several of the observations that we report here fit this trend. In SLIs, E‐Cadherin, β‐Catenin, and F‐Actin demonstrate reduced distributions in CMT1A but are increased or unchanged in HNPP, whereas Connexin29 exhibits dramatically increased distribution in CMT1A and less so in HNPP. At Nodes of Ranvier, Kv1.2 distribution is reduced at juxtaparanodes and is spread internodally in CMT1A more so than in HNPP; Nav demonstrates nodal widening (increased distribution) in CMT1A but is unchanged in HNPP, and Caspr demonstrates potentially more internodal spread in CMT1A than in HNPP. These results suggest that changes at Nodes of Ranvier, such as nodal widening and disrupted domain‐specific molecular patterning, along with accompanying abnormalities at SLIs, likely impair proper functionality of these key myelin domains, with more severe impairments in CMT1A than HNPP. These architectural alterations are predicted to compromise metabolic support and alter axonal excitability despite grossly intact compact myelin. Created in https://BioRender.com.

Together, these findings suggest that the molecular and organizational defects identified in CMT1A and HNPP can arise independently of overt demyelination and likely reflect early, intrinsic disturbances in molecular myelin domain architecture rather than secondary consequences of myelin loss. These alterations may therefore represent early and persistent pathogenic mechanisms that contribute to disease progression, with the degree of disruption aligning with the relative severity of the corresponding mouse models, more pronounced in CMT1A than in HNPP (summarized in Figure 8).

## 4 Discussion

Although secondary axonal degeneration has historically been proposed as the primary driver of functional decline in CMT1A (Krajewski et al. 2000; Manganelli et al. 2016), recent evidence from rodent models, including our own, has challenged this view by demonstrating significant neuromuscular impairment in the absence of overt axonal loss (Moss et al. 2022; Verhamme et al. 2011; Robertson et al. 2002). These findings have motivated a reexamination of disease mechanisms, particularly focusing on the possibility that primary dysfunction of myelin itself contributes directly to disease progression in both CMT1A and HNPP. Given that both disorders result from copy number variation of *PMP22*, encoding a myelin‐enriched transmembrane protein with structural similarity to Claudins, and that previous studies have implicated PMP22 in cell adhesion, we hypothesized that PMP22 regulates molecular architecture within peripheral myelin through junctional mechanisms.

Further support for a Claudin‐like function of PMP22 comes from the observation that several PMP22 missense mutations causing CMT Type 1E (CMT1E) occur within or adjacent to ECL1 of PMP22, a region structurally analogous to the Claudin ECL1 domain implicated in intercellular adhesion (Bao et al. 2025; Venugopal et al. 2019). This includes residues within the Claudin‐like motif of PMP22 (W28…D37‐L38‐W39…C42…C53), with reported pathogenic substitutions p.Trp28Arg, p.Asp37Val, and p.Trp39Cys (Fabrizi et al. 1999; Taioli et al. 2012; Boerkoel et al. 2002). Moreover, recent structural modeling and *in silico* mutagenesis of PMP22 have provided new insights into how CMT1E‐associated variants may perturb local folding, helix–helix packing and transmembrane topology (Mittendorf et al. 2014; Pashkova et al. 2024; Ward et al. 2025). These structural advances establish a framework for future studies to directly test whether mutations in the Claudin‐like motif impair PMP22 stability or its adhesive interactions within compact myelin.

Motivated by these structural insights and building on prior qualitative reports demonstrating that the organization of E‐Cadherin, MAG and Kv1.1 are altered in myelin from *PMP22^+/−^* and *PMP22^–^^/−^* mice (Guo et al. 2014; Neuberg et al. 1999), we employed high‐resolution confocal imaging and quantitative analysis of teased peripheral nerve fibers from constitutive CMT1A and HNPP model mice. Our data extend these prior qualitative observations in HNPP by introducing quantitative imaging analyses that reveal measurable, protein‐specific alterations in SLI molecular organization in CMT1A and HNPP fibers exhibiting largely preserved compact myelin. Importantly, the increased sensitivity of our dual‐metric approach led us to distinct conclusions about junctional and MAG‐associated abnormalities in HNPP: whereas Hu et al. (2016) reported approximately 15% of SLIs abnormal for E‐Cadherin and about 5% for MAG at 3 months, our quantitative assessment detected no significant changes in E‐Cadherin but identified far more pronounced alterations in MAG distribution and mean intensity.

Collectively, our results demonstrate that *PMP22* dosage critically regulates the molecular architecture of SLIs, leading to widespread disruption of adherens junction components, E‐Cadherin, β‐Catenin, p120‐Catenin, and F‐Actin, with divergent changes in CMT1A and HNPP. In CMT1A, these proteins exhibited a compacted or reduced distribution, whereas in HNPP, some distributions were increased, including F‐Actin. These findings suggest that *PMP22* dosage bidirectionally alters junctional organization and SLI morphology, potentially destabilizing Schwann cell cytoskeletal architecture. Interestingly, not all SLI‐associated proteins were equally affected. CADM4 remained unchanged, while Connexin29 and MAG exhibited significant changes in both models, often with more dramatic distribution changes in CMT1A. Notably, Cx29 also displayed focal accumulations along internodes in CMT1A nerves, suggesting misregulation beyond the SLI compartment. Additionally, mean Cx32 intensity was reduced at SLIs in both CMT1A and HNPP to a similar degree. It is well established that *Cx32/GJB1*‐null mice (CMT1X model) exhibit CMT‐like neuropathy with slowed conduction and progressive myelin defects (Nelles et al. 1996; Anzini et al. 1997), whereas *MAG*‐null mice (Montag et al. 1994; Weiss et al. 2001) display related myelin and functional abnormalities and have been suggested to mimic aspects of HNPP (Yin et al. 1998). Conditional deletion of E‐Cadherin in Schwann cells (Young et al. 2002) causes peripheral nerve structural alterations, although functional consequences have not been reported. In contrast, *Cx29*‐null mice show no overt peripheral neuropathy under baseline conditions (Eiberger et al. 2006), but compensatory expression and/or function of other connexins has not been systematically examined. The convergence of these phenotypes underscores the functional relevance of these junctional components and supports the concept that myelin adhesion and gap‐junctional communication are integral to Schwann‐cell integrity and axonal support. These selective alterations point to protein‐specific regulatory effects of PMP22 on myelin architecture and highlight the possibility that PMP22 coordinates the organization of multiple molecular domains within the myelin sheath through potentially distinct mechanisms.

Given the anatomical linkage between SLIs and the Node of Ranvier via the inner mesaxon and known associations between Cx29 and Kv1 channels (Rash et al. 2016), we also assessed nodal subdomain organization. Consistent with a model of impaired axo‐glial communication, we observed disrupted patterning of Node of Ranvier axonal components including Nav channels, Caspr, and Kv1.2 channels in both CMT1A and HNPP, with more severe changes frequently observed in CMT1A. These included nodal widening, loss of juxtaparanodal Kv1.2 enrichment and abnormal spreading of Kv1.2 and Caspr along internodes. Because molecular patterning at Nodes of Ranvier is essential for saltatory conduction (Scherer and Arroyo 2002; Rasband and Peles 2025), this disorganization is expected to compromise conduction velocity and excitability. Future studies are needed to test this prediction using electrophysiological and pharmacologic approaches.

When interpreting these findings, several considerations are important. First, the CMT1A and HNPP models used here were generated on different genetic backgrounds (C57BL/6J and 129S1/SvImJ, respectively), reflecting model availability rather than experimental design. Second, nodal analyses in HNPP nerves may be influenced by tomacular remodeling, which could secondarily affect local molecular organization. Third, our analyses necessarily focused on intact fibers that remain preserved during teasing, and average G‐Ratios measured in these fibers were largely unchanged, indicating grossly preserved compact myelin within the analyzed subset. The absence of prominent segmental demyelination in our teased‐fiber preparations may also reflect the possibility that segmentally demyelinated regions are more fragile and thus more prone to breakage during teasing (Bolon et al. 2020; Krinke et al. 2000), potentially limiting their retention in the final dataset. Importantly, despite these considerations, the consistency of junctional and nodal phenotypes across both models strongly supports a shared role for *PMP22* dosage in regulating myelin architecture.

Together, our findings support a model in which PMP22 functions as a structural organizer of myelin, coordinating E‐Cadherin‐based adherens complexes and Node of Ranvier ion channel patterning that together are predicted to maintain Schwann‐cell architecture, axo‐glial coupling and efficient nerve conduction. This aligns with prior evidence that PMP22 may interact with E‐Cadherin in myelin (Guo et al. 2014) and that loss of E‐Cadherin disrupts autotypic junction formation and myelin integrity (Tricaud et al. 2005; Young et al. 2002). This model reframes the pathogenesis of CMT1A and HNPP as diseases rooted in molecular disorganization of myelin architecture, challenging the prevailing view that secondary axonal degeneration is the exclusive driver of disability. It also provides a mechanistic basis for why compact myelin may appear morphologically preserved while still being functionally impaired. Moreover, the inverse or phenotype severity‐correlated changes observed in CMT1A and HNPP strengthen the argument that *PMP22* dosage has a direct and bidirectional impact on Schwann cell structure and function. From a therapeutic perspective, these findings highlight junctional complexes, particularly adherens junction components and their cytoskeletal partners, as actionable molecular targets. Based on our findings, strategies aimed at stabilizing or restoring junctional organization would be predicted to improve conduction properties and metabolic support in CMT1A and HNPP, even in the absence of myelin regeneration. Furthermore, this work offers a conceptual framework for investigating related junctional pathomechanisms in other inherited dysmyelinating and acquired demyelinating neuropathies.

In this context, disruption of PMP22‐mediated adhesion should be viewed as one of several possible mechanisms contributing to myelin dysfunction rather than the sole initiating event. PMP22 deficiency or overexpression could perturb membrane trafficking, lipid organization, or protein turnover, indirectly affecting junctional stability and axo‐glial signaling. Alternatively, junctional disorganization may arise as a compensatory response to altered Schwann cell homeostasis or cytoskeletal remodeling. Future studies integrating proteomic, lipidomic, live imaging, and electron microscopy approaches will be essential to determine whether junctional instability represents a primary or secondary event in disease progression.

Overall, our data support a model in which aberrant PMP22‐dependent molecular organization disrupts the coordinated compartmentalization required for efficient myelin function. This may impair essential processes such as metabolic coupling and axonal ion homeostasis, though these mechanisms remain to be formally tested. These findings advance our understanding of how structural disorganization may underlie functional deficits in peripheral neuropathy and set the stage for mechanistic studies to define causal relationships and identify therapeutic strategies that can restore myelin domain integrity. Future work should evaluate whether emerging *PMP22*‐targeted and agnostic therapies (Bai et al. 2022; Zhao et al. 2018; Stavrou and Kleopa 2023; Ha et al. 2020; Krauter et al. 2024), currently in various stages of preclinical and clinical development can ameliorate these molecular phenotypes.

## Author Contributions

K.R.M. designed the study, performed experiments and data analysis, and wrote the manuscript. M.A.A. and D.R.G. performed experiments and data analysis. A.H. assisted with experimental design and manuscript writing and editing. All authors approved the submitted version of the manuscript.

## Funding

This work was supported by an NIH K22 Award (K22NS125057) and a Johns Hopkins Merkin Peripheral Neuropathy and Nerve Regeneration Center Grant to K.R.M.

## Ethics Statement

Animal experiments were conducted with approval from the Johns Hopkins University (approval number Mo21M332) and University of Missouri (approval number 57681) Animal Care and Use Committee. All other experiments were conducted with approval from the University of Missouri Institutional Biosafety Committee (approval number 21140).

## Conflicts of Interest

The authors declare no conflicts of interest.

## Supporting information


**Data S1:** Supporting information.

## Data Availability

The data that support the findings of this study are available from the corresponding author upon reasonable request.
