# Supplementary material for: Aberrant Molecular Myelin Architecture in Charcot–Marie–Tooth Disease Type 1A and Hereditary Neuropathy With Liability to Pressure Palsies
Source: Glia. 2025 Dec 16;74(2):e70124. doi: 10.1002/glia.70124 (PMC12706824; doi:10.1002/glia.70124)
Supplement: Supplementary file 1 — Data S1: Supporting information. [file GLIA-74-0-s001.pdf]

Supplemental Figures and Legends

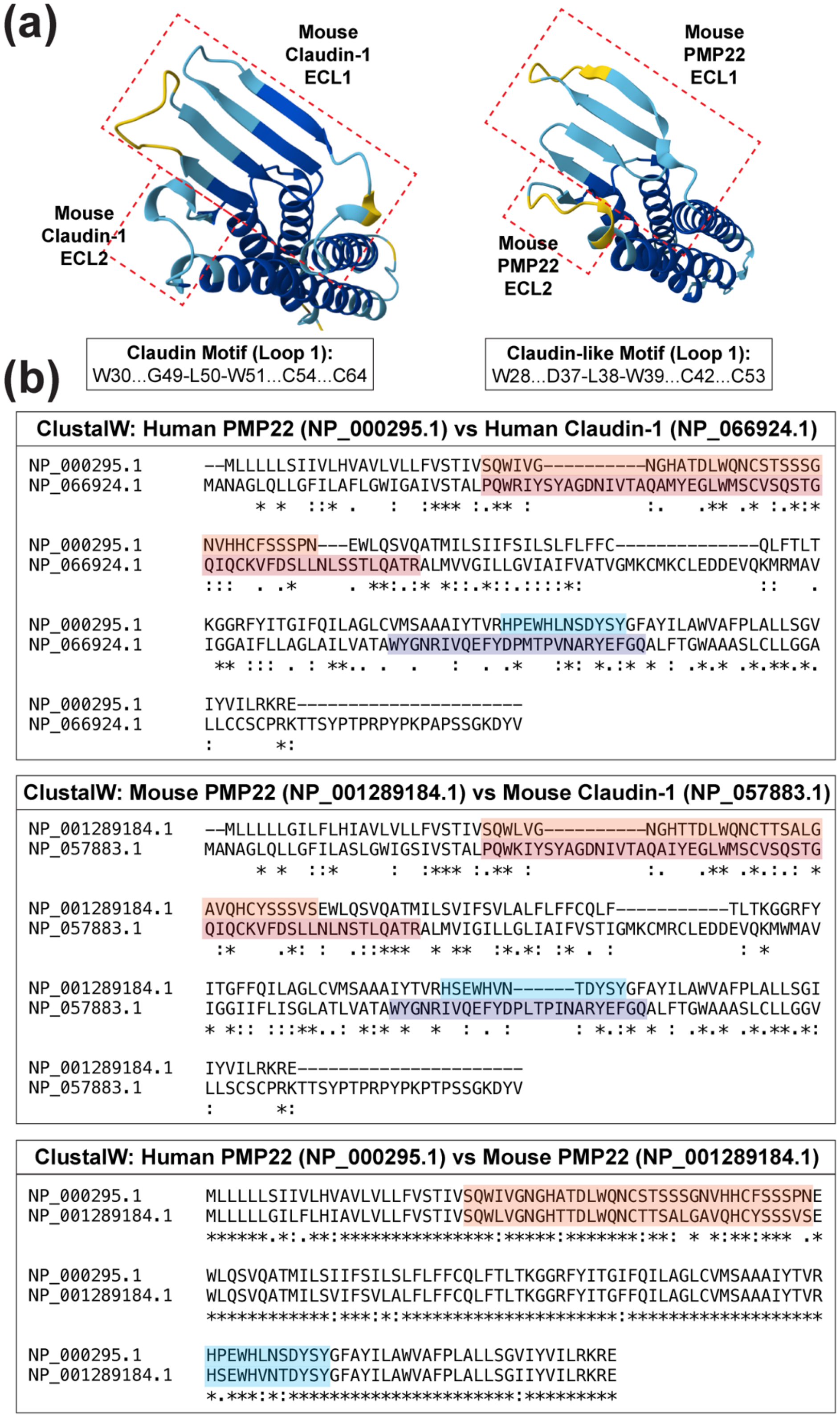

Supplemental Figure 1. PMP22 Exhibits Structural Similarity to Claudin-1 Despite Modest Sequence Identity. (a) AlphaFold predicted structures of mouse Claudin-1 and mouse PMP22 extracellular loops (ECLs) viewed from above (AlphaFold Protein Structure Database). The structural similarity between the ECLs is

striking supporting the notion that PMP22 functions similarly to Claudin proteins. **(b)** ClustalW alignments of human PMP22 with human Claudin-1 (top), mouse PMP22 with mouse Claudin-1 (middle) and human PMP22 with mouse PMP22 (bottom). PMP22 is highly conserved from mouse to human but sequence similarity and identity between PMP22 and Claudin-1 is more limited. The extracellular loop 1 (ECL1) sequences for PMP22 and Claudin-1 are highlighted in orange/red and the extracellular loop 2 (ECL2) sequences for PMP22 and Claudin-1 are highlighted in blue/purple.

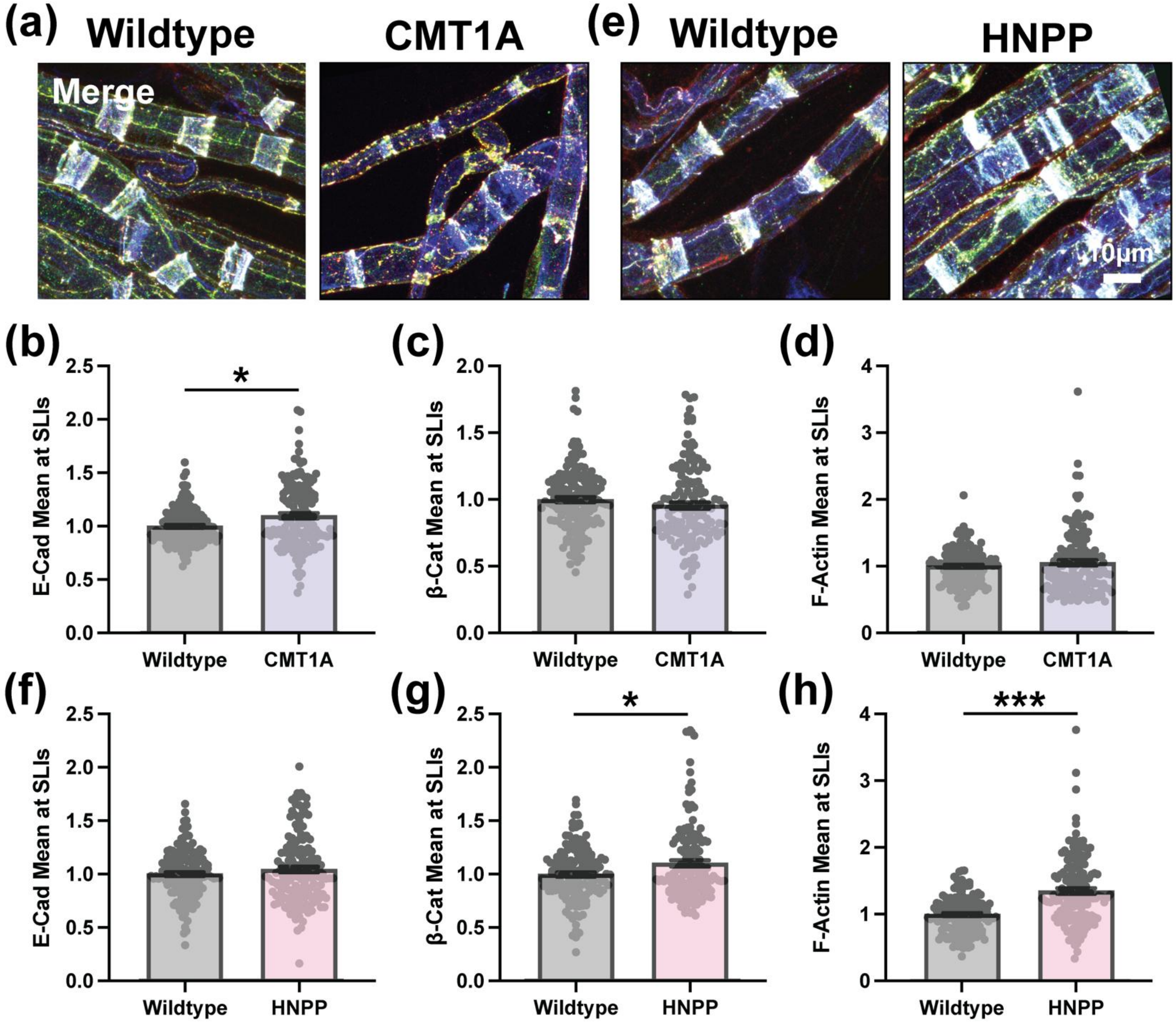

Supplemental Figure 2. Subtle Alterations in Adherens Junction and F-Actin Mean Intensity at SLIs in CMT1A and HNPP Model Myelin. Representative images of 3-month-old **(a)** WT (C57BL/6J) and CMT1A or **(e)** WT (129S1/SvImJ) and HNPP teased tibial nerve fibers stained for the adherens junction proteins E-Cadherin (red), β-Catenin (green) and F-Actin (blue) merged. Quantification of mean signal intensity at SLIs for **(b, f)** E-Cadherin, **(c, g)** β-Catenin and **(d, h)** F-Actin in CMT1A and HNPP, respectively. n=5 animals (~30 SLIs/animal). Bar graphs represent mean ± SEM with individual data points. Datasets were analyzed with three complementary statistical approaches (unpaired t-test with all individual data points, unpaired t-test with experimental means and nested t-test). ***$p<0.05$ demonstrate statistical significance with all three t-test statistics and *$p<0.05$ demonstrate statistical significance with a single t-test statistic. Scale bar, 10µm.

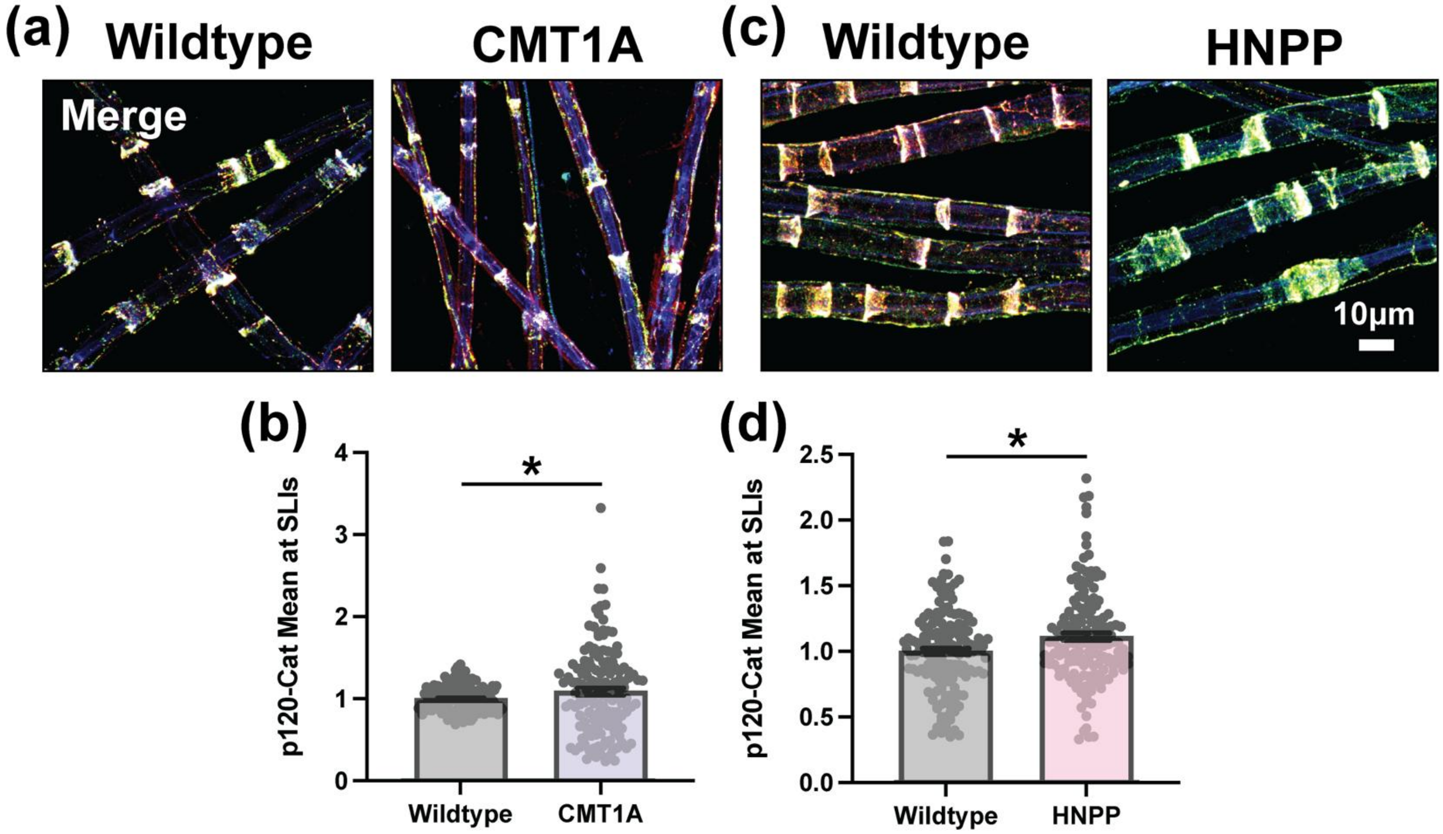

Supplemental Figure 3. Modest Increase in p120-Catenin Mean Intensity at SLIs in CMT1A and HNPP Model Peripheral Nerve Myelin. Representative images of 3-month-old **(a)** WT (C57BL/6J) and CMT1A or **(c)** WT (129S1/SvImJ) and HNPP teased tibial nerve fibers stained for the adherens junction proteins E-Cadherin (red), p120-Catenin (green) and F-Actin (blue) merged. Quantification of mean p120-Catenin signal intensity at SLIs in **(b)** CMT1A and **(d)** HNPP. n=5 animals (~30 SLIs/animal). Bar graphs represent mean ± SEM with individual data points. Datasets were analyzed with three complementary statistical approaches (unpaired t-test with all individual data points, unpaired t-test with experimental means and nested t-test). *$p<0.05$ demonstrate statistical significance with a single t-test statistic. Scale bar, 10μm.

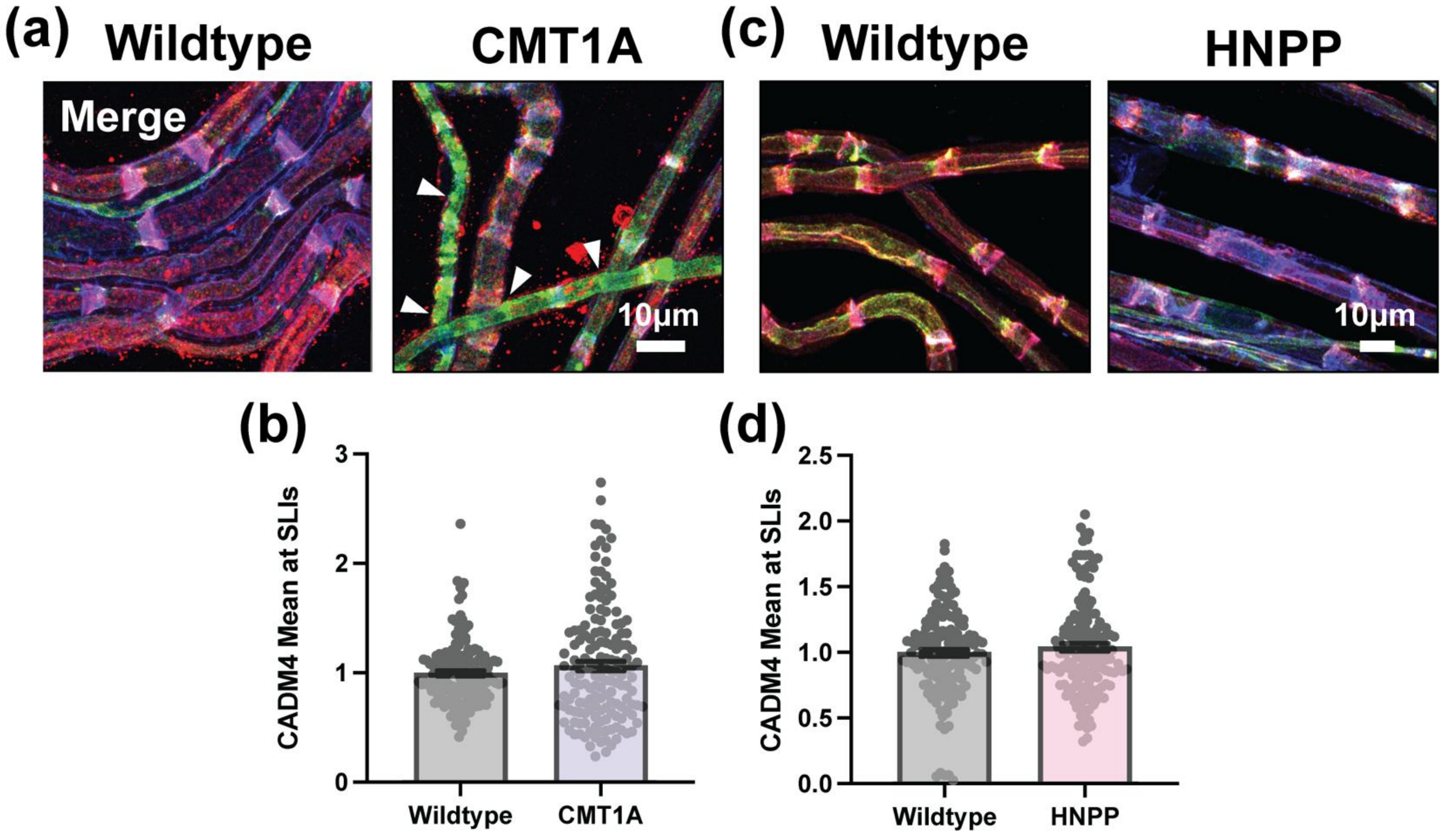

Supplemental Figure 4. Unchanged CADM4 Mean Intensity at SLIs in CMT1A and HNPP Mouse Model Myelin. Representative images of 3-month-old **(a)** WT (C57BL/6J) and CMT1A or **(c)** WT (129S1/SvImJ) and HNPP teased tibial nerve fibers stained for CADM4 (red), Connexin29 (green) and F-Actin (blue) merged. Note the focal accumulations of Cx29 outside the SLI compartment in CMT1A model myelin (arrowheads). Quantification of mean CADM4 signal intensity at SLIs in **(b)** CMT1A and **(d)** HNPP. n=5 animals (~30 SLIs/animal). Bar graphs represent mean ± SEM with individual data points. Datasets were analyzed with three complementary statistical approaches (unpaired t-test with all individual data points, unpaired t-test with experimental means and nested t-test). All analyses were not significant ($p>0.05$). Scale bars, 10µm.

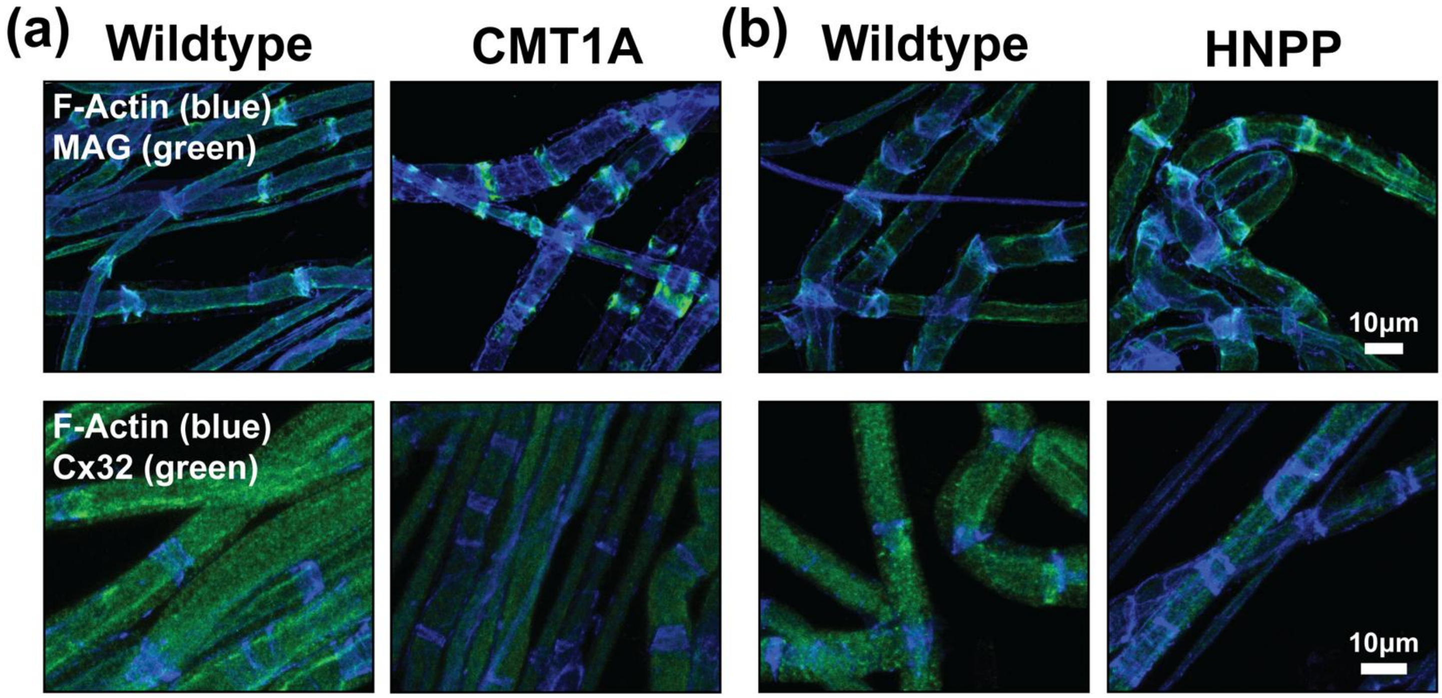

Supplemental Figure 5. MAG and Connexin32 Merged Images. Representative images of 3-month-old **(a)** WT (C57BL/6J) and CMT1A or **(b)** WT (129S1/SvImJ) and HNPP teased tibial nerve fibers stained for *(top panel)* MAG (green) and F-Actin (blue) merged and *(bottom panel)* Connexin32 (green) and F-Actin (blue) merged. Scale bars, 10µm.

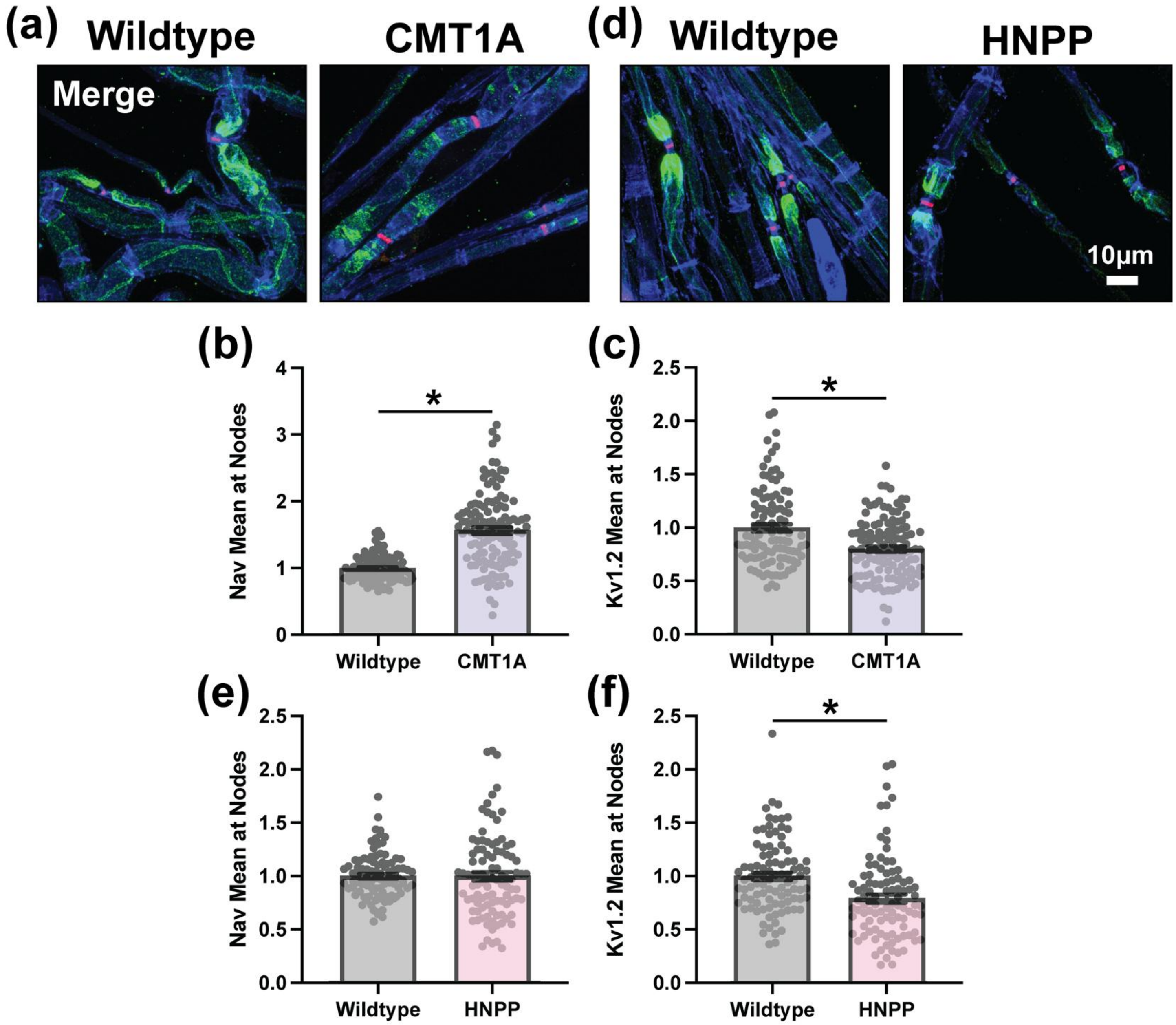

Supplemental Figure 6. Altered Ion Channel Mean Intensity at Nodes of Ranvier in CMT1A and HNPP Model Myelin. Representative images of 3-month-old **(a)** WT (C57BL/6J) and CMT1A or **(d)** WT (129S1/SvImJ) and HNPP teased tibial nerve fibers stained with anti-Pan Nav (red), anti-Kv1.2 (green) and F-Actin (blue) merged. Quantification of mean signal intensity at Nodes of Ranvier for **(b, e)** Nav and **(c, f)** Kv1.2 in CMT1A and HNPP, respectively. n=5 animals (~15-25 nodes/animal). Bar graphs represent mean ± SEM with individual data points. Datasets were analyzed with three complementary statistical approaches (unpaired t-test with all individual data points, unpaired t-test with experimental means and nested t-test). *$p<0.05$ demonstrate statistical significance with a single t-test statistic. Scale bar, 10μm.

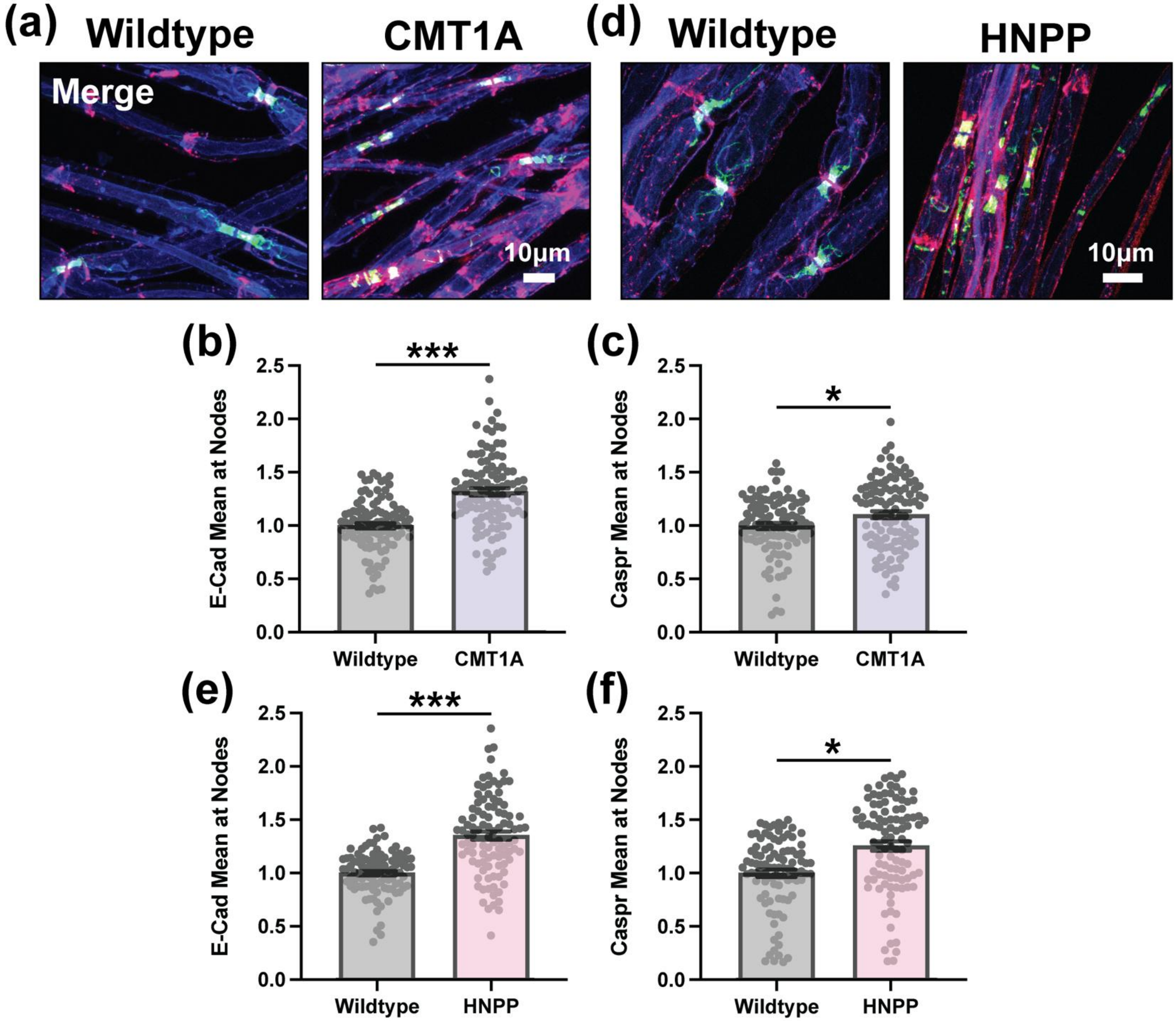

Supplemental Figure 7. Increased Caspr and E-Cadherin Mean Intensity at Nodes of Ranvier in CMT1A and HNPP Model Peripheral Nerve Myelin. Representative images of 3-month-old **(a)** WT (C57BL/6J) and CMT1A or **(d)** WT (129S1/SvImJ) and HNPP teased tibial nerve fibers stained for E-Cadherin (red), Caspr (green) and F-Actin (blue) merged. Quantification of mean signal intensity at Nodes of Ranvier for **(b, e)** E-Cadherin and **(c, f)** Caspr in CMT1A and HNPP, respectively. n=5 animals (~15-25 nodes/animal). Bar graphs represent mean ± SEM with individual data points. Datasets were analyzed with three complementary statistical approaches (unpaired t-test with all individual data points, unpaired t-test with experimental means and nested t-test). ***$p<0.05$ demonstrate statistical significance with all three t-test statistics and *$p<0.05$ demonstrate statistical significance with a single t-test statistic. Scale bars, 10μm.

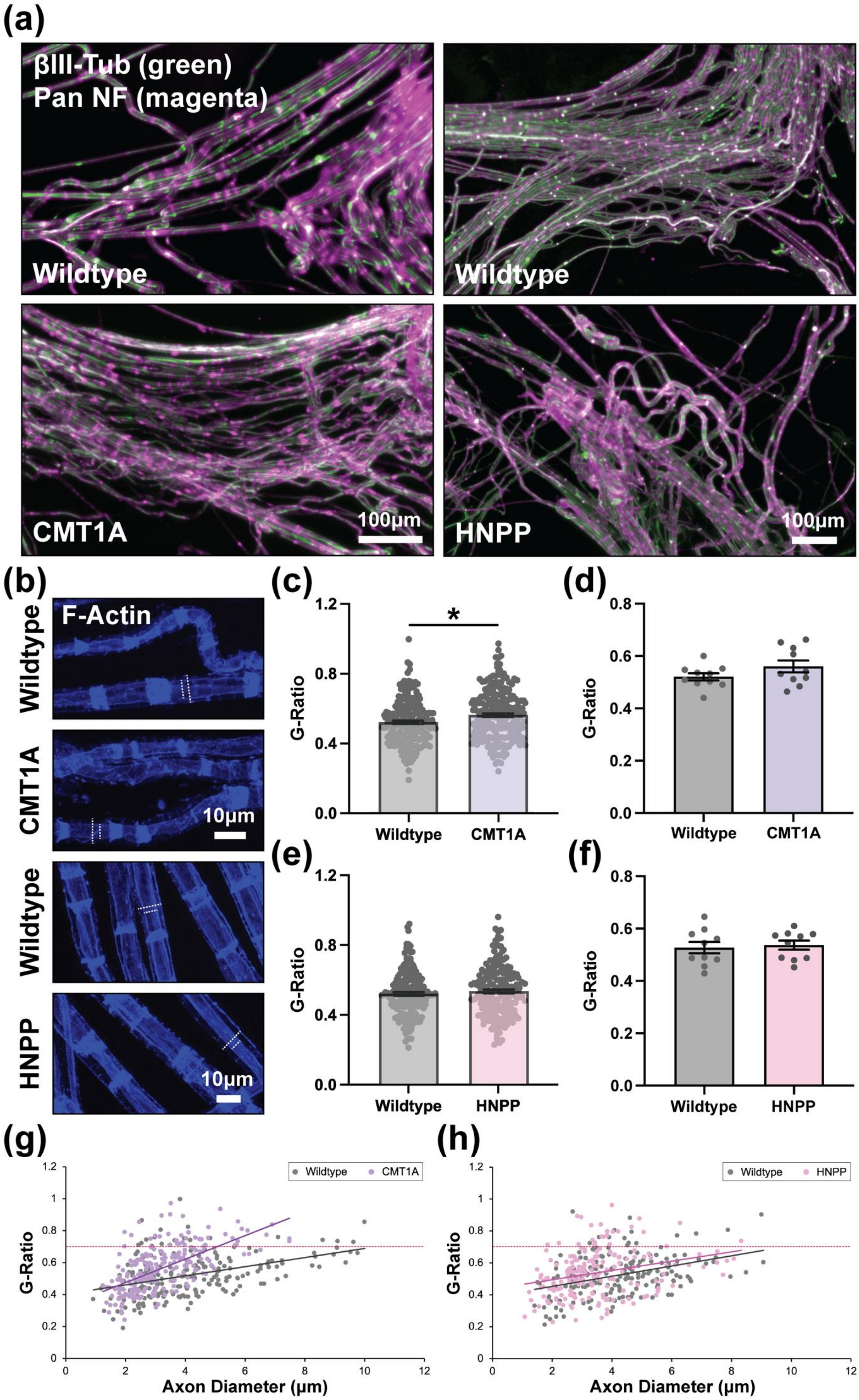

(a)
βIII-Tub (green)
Pan NF (magenta)
Wildtype
Wildtype
CMT1A
100μm
HNPP
100μm
(b)
F-Actin
Wildtype
CMT1A
10μm
Wildtype
HNPP
10μm
(c)
G-Ratio
Wildtype
CMT1A
*
(d)
G-Ratio
Wildtype
CMT1A
(e)
G-Ratio
Wildtype
HNPP
(f)
G-Ratio
Wildtype
HNPP
(g)
Wildtype
CMT1A
G-Ratio
Axon Diameter (μm)
(h)
Wildtype
HNPP
G-Ratio
Axon Diameter (μm)

Supplemental Figure 8. Overall Preservation of Compact Myelin in Analyzed Fibers from 3-Month-Old CMT1A and HNPP Model Mice. (a) Representative widefield fluorescence images of 3-month-old WT (C57BL/6J) and CMT1A or WT (129S1/SvImJ) and HNPP teased tibial nerve fibers stained for βIII-Tubulin (green) and pan Neurofascin (magenta) merged. Note that segmentally demyelinated regions are not abundant. Scale bars, 100μm. **(b)** G-Ratios were calculated in a subset of our images by measuring the axon diameter (shorter white dashed line) as labeled by F-Actin (blue) and using the total fiber diameter measured previously for distribution calculations (longer white dashed line). Scale bars, 10μm. Quantification of average G-Ratios as displayed by **(c,e)** bar graphs ± SEM with all individual data points and **(d,f)** bar graphs ± SEM with experimental means. n=10 animals (~10-30 fibers/animal). Datasets were analyzed unpaired t-test either with all individual data points or experimental means aligning with the displayed graph. $^*p<0.05$ denotes statistical significance. Plots of G-ratio against axon diameter with trendlines for **(g)** CMT1A and **(h)** HNPP as compared to WT were created with the same data. Normal G-Ratio (0.7) is denoted with a red dashed line.

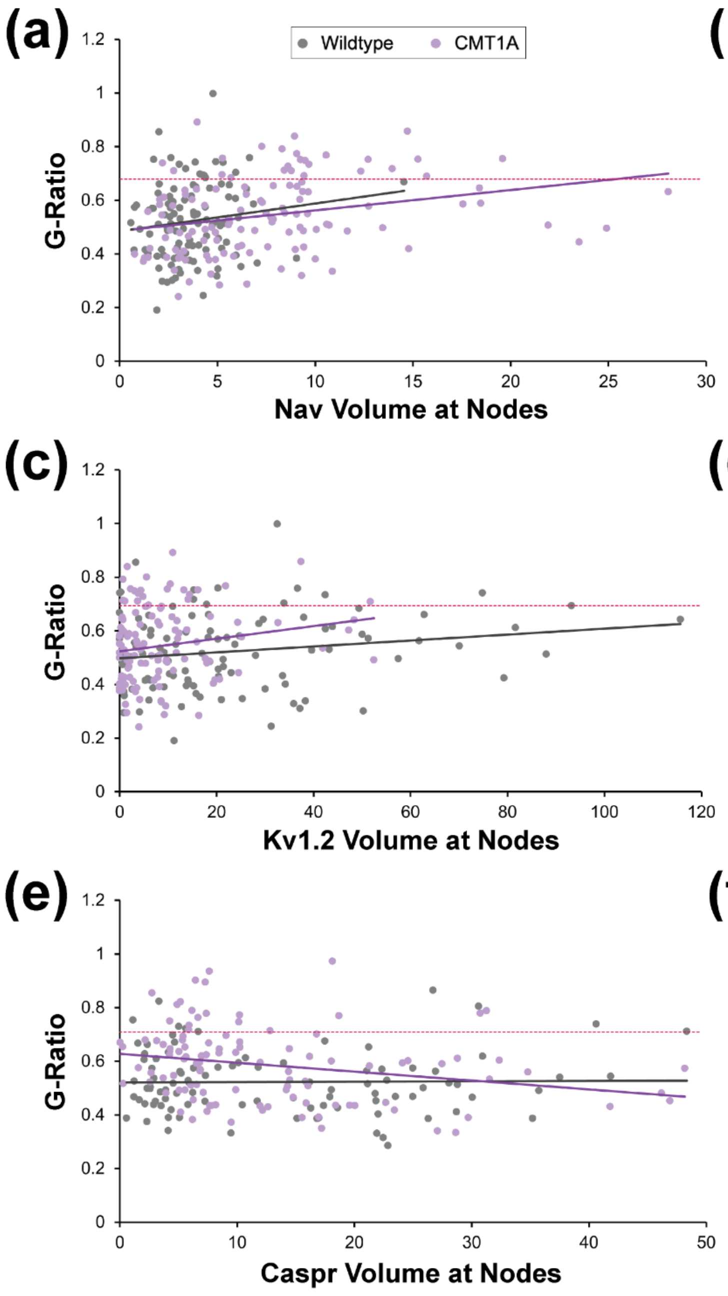

Supplemental Figure 9. Severity of Nodal Defects Does Not Strongly Correlate with G-Ratio. Plots of G-ratio against the following protein distributions at Nodes of Ranvier: **(a,b)** Nav, **(c,d)** Kv1.2 and **(e,f)** Caspr in CMT1A and HNPP as compared to WT, respectively. Trendlines are shown for each dataset and normal G-Ratio (0.7) is denoted with a red dashed line. Note the absence of an enrichment of elevated G-Ratios among fibers exhibiting the more severe nodal phenotypes (increased Nav and Caspr distributions and reduced Kv1.2 distribution).

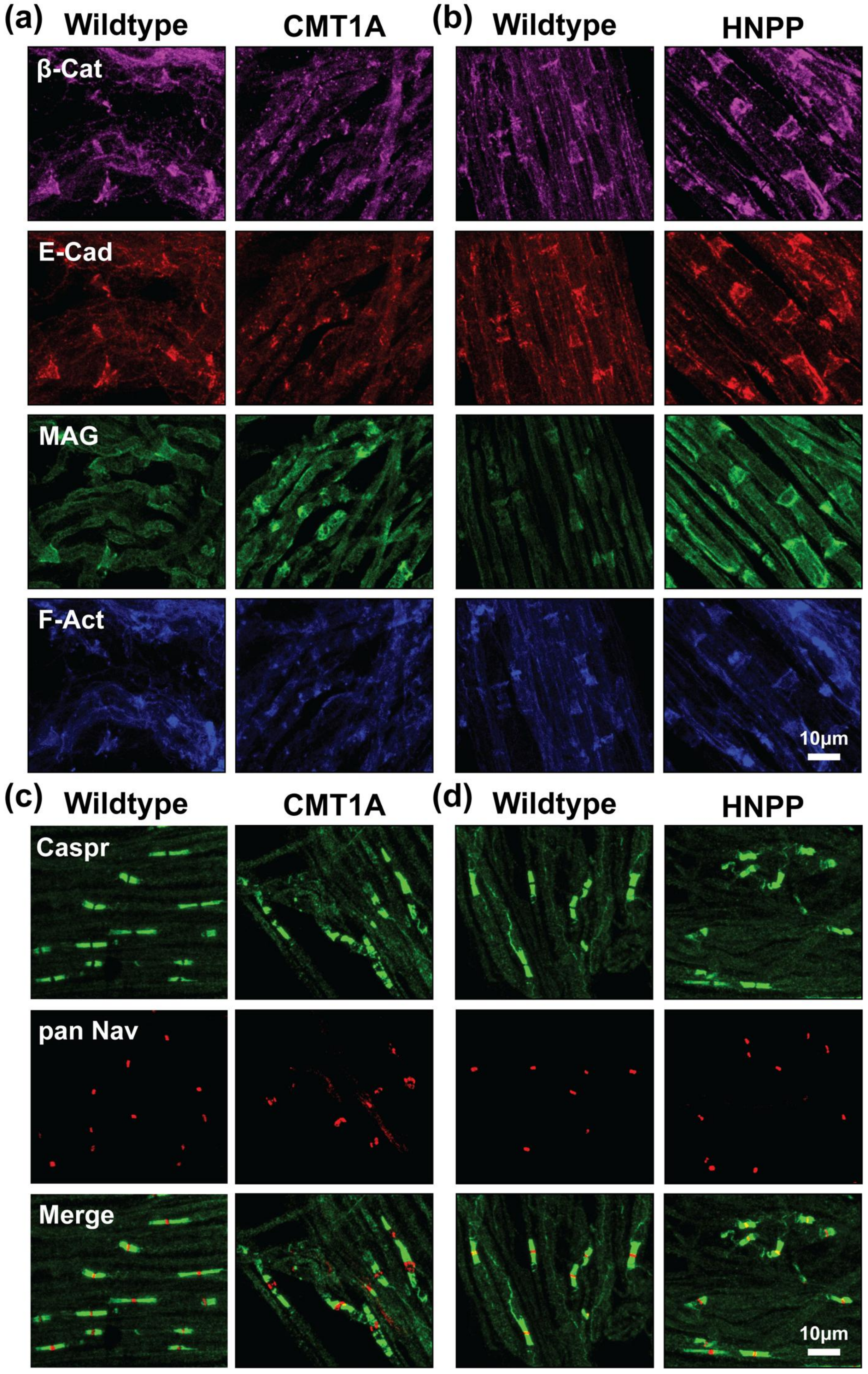
(a)
Wildtype
CMT1A
(b)
Wildtype
HNPP
β-Cat
E-Cad
MAG
F-Act
10μm
(c)
Wildtype
CMT1A
(d)
Wildtype
HNPP
Caspr
pan Nav
Merge
10μm

Supplemental Figure 10. Qualitatively Comparable Disruption of Molecular Myelin Architecture in Developing Peripheral Nerves from CMT1A and HNPP Model Mice. Representative images of postnatal day 15 (P15) **(a,c)** WT (C57BL/6J) and CMT1A or **(b,d)** WT (129S1/SvImJ) and HNPP teased tibial nerve fibers stained for **(a,b)** β-Catenin (magenta), E-Cadherin (red), MAG (green) and F-Actin (blue) at SLIs and **(c,d)** Caspr (green) and pan Nav (red) with merge at Nodes of Ranvier. Scale bars, 10μm.
